# Precisely Activating cGAS‐STING Pathway with a Novel Peptide‐Based Nanoagonist to Potentiate Immune Checkpoint Blockade Cancer Immunotherapy

**DOI:** 10.1002/advs.202309583

**Published:** 2024-01-17

**Authors:** Yumeng Xing, Ao Peng, Jianhui Yang, Zhifei Cheng, Yi Yue, Feilong Liu, Fenghe Li, Yang Liu, Qi Liu

**Affiliations:** ^1^ School of Pharmacy Anhui Medical University Hefei 230032 China; ^2^ College of Chemistry and Chemical Engineering Xiamen University Xiamen 361005 China; ^3^ School of Pharmacy Anhui University of Chinese Medicine Hefei 230012 China; ^4^ College of Chemistry Nankai University Tianjin 300071 China

**Keywords:** antimicrobial peptides, cancer immunotherapy, cGAS‐STING activation, multi‐stimuli activatable, PD‐1/PD‐L1 blockade

## Abstract

As an essential intracellular immune activation pathway, the cGAS‐STING pathway has attracted broad attention in cancer treatment. However, low bioavailability, nonspecificity, and adverse effects of small molecule STING agonists severely limit their therapeutic efficacy and in vivo application. In this study, a peptide‐based STING agonist is first proposed, and KLA is screened out to activate the cGAS‐STING pathway by promoting mitochondrial DNA (mtDNA) leakage. To precisely activate the cGAS‐STING pathway and block the PD‐1/PD‐L1 pathway, a multi‐stimuli activatable peptide nanodrug (MAPN) is developed for the effective delivery of KLA and PD‐L1 antagonist peptide (CVR). With rational design, MAPN achieved the site‐specific release of KLA and CVR in response to multiple endogenous stimuli, simultaneously activating the cGAS‐STING pathway and blocking PD‐1/PD‐L1 pathway, ultimately initiating robust and durable T cell anti‐tumor immunity with a tumor growth inhibition rate of 78% and extending the median survival time of B16F10 tumor‐bearing mice to 40 days. Overall, antimicrobial peptides, which can promote mtDNA leakage through damaging mitochondrial membranes, may be potential alternatives for small molecule STING agonists and giving a new insight for the design of novel STING agonists. Furthermore, MAPN presents a universal delivery platform for the effective synergy of multiple peptides.

## Graphical abstract

Antimicrobial peptides are proposed to activate the cGAS‐STING pathway by promoting mitochondrial DNA leakage, providing a new insight for the design of novel STING agonists. Furthermore, a multi‐stimuli activatable peptide nanodrug is designed for site‐specific delivery of different peptides, simultaneously activating the cGAS‐STING pathway and blocking PD‐1/PD‐L1 pathway, ultimately initiating robust and durable T cell anti‐tumor immunity.

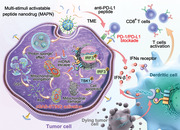

## 1 Introduction

The cyclic guanosine monophosphate (GMP)‐adenosine monophosphate synthase (cGAS)‐stimulator of interferon genes (STING) pathway has been identified as a critical role in initiating innate and adaptive anti‐tumor immune responses.^[^ ^1^ ^]^ Serving as a sensor protein for virus DNA, the cGAS enzyme can sense exogenous DNA fragments in the cytoplasm and catalyze adenylate triphosphate and guanosine triphosphate into 2′,3′‐cyclic GMP‐adenosine monophosphate (cGAMP) to activate STING. Activated STING would further activate tank‐binding kinase 1 (TBK1) and interferon regulatory factor 3 (IRF3) to dramatically elevate the secretion levels of type I interferon (IFN‐I) and other proinflammatory cytokines, promoting the presentation of tumor‐associated antigens by dendritic cells (DCs) and eventually activating T cell anti‐tumor immunity.^[^ ^1^, ^2^ ^]^ The activation of the cGAS‐STING pathway is beneficial for reversing the immunosuppressive tumor microenvironment (TME), potentially making patients more susceptible to immune checkpoint blockade (ICB) immunotherapy, such as programmed death ligand 1/programmed death ligand 1 (PD‐1/PD‐L1) pathway blockade.^[^ ^3^ ^]^ In recent years, several STING agonists are under clinical trials or have been applied clinically to combat various malignant tumors.^[^ ^4^ ^]^ However, current small molecule STING agonists (e.g., cyclic dinucleotide (CDN), metal ions, DNA‐damaged chemo drugs, and small molecule inhibitors) usually suffer from low bioavailability, nonspecificity, and serious adverse effects.^[^ ^4^, ^5^ ^]^ Therefore, more efforts should be made to develop novel STING agonists for more safe and effective cancer immunotherapy.

Compared to small‐molecule drugs, peptides usually have higher activity and selectivity due to the higher affinity with targets, making them powerful supplements to small‐molecule drugs.^[^ ^6^ ^]^ Peptides also exhibit low toxicity because their metabolites are amino acids, which usually have no obvious side effects on the human body.^[^ ^6c^ ^]^ Antimicrobial peptides (AMPs) are a class of natural cationic peptides with amphipathic structures, typically containing 5–50 amino acid residues.^[^ ^7^ ^]^ Recently, AMPs have attracted broad attention in cancer treatment, as its potential to selectively kill cancer cells through a non‐receptor‐mediated membranolytic mechanism.^[^ ^8^ ^]^ In addition, due to the highly negative transmembrane potential of the mitochondrial membrane,^[^ ^9^ ^]^ a proportion of AMPs can quickly damage the mitochondrial membrane, leading to mitochondrial DNA (mtDNA) leakage into the cytoplasm.^[^ ^10^ ^]^ The leaked mtDNA would be sensed by the cGAS enzyme and ultimately activate the cGAS‐STING pathway.^[^ ^11^ ^]^ Given the advantage of peptides, AMPs might be excellent candidates for STING agonists. Compared to anti‐PD‐L1 antibodies, synthetic PD‐L1 antagonist peptides also have higher stability, reduced immunogenicity, and lower production costs.^[^ ^12^ ^]^ Additionally, peptides are easier to simulate the interaction between PD‐1 and PD‐L1 than small molecules.^[^ ^6c^ ^]^ Obviously, the combination of AMPs and PD‐L1 antagonist peptides would further unleash the T cell anti‐tumor immunity. However, from the perspective of drug delivery, cationic AMPs, and PD‐L1 antagonist peptides are easily degraded and cleared by the reticulo‐endothelial system (RES) after systemic administration.^[^ ^12b^ ^]^ Furthermore, most AMPs hardly cross cell membrane and escape from endosomes, which severely limits their bioactivity in tumor inhibition and cGAS‐STING pathway activation.^[^ ^13^ ^]^ Thus, it is necessary to design a feasible strategy to deliver PD‐L1 antagonist peptides and AMPs into specific action sites (tumor extracellular and intracellular) while maintaining low normal tissue toxicity.

**Scheme 1:**
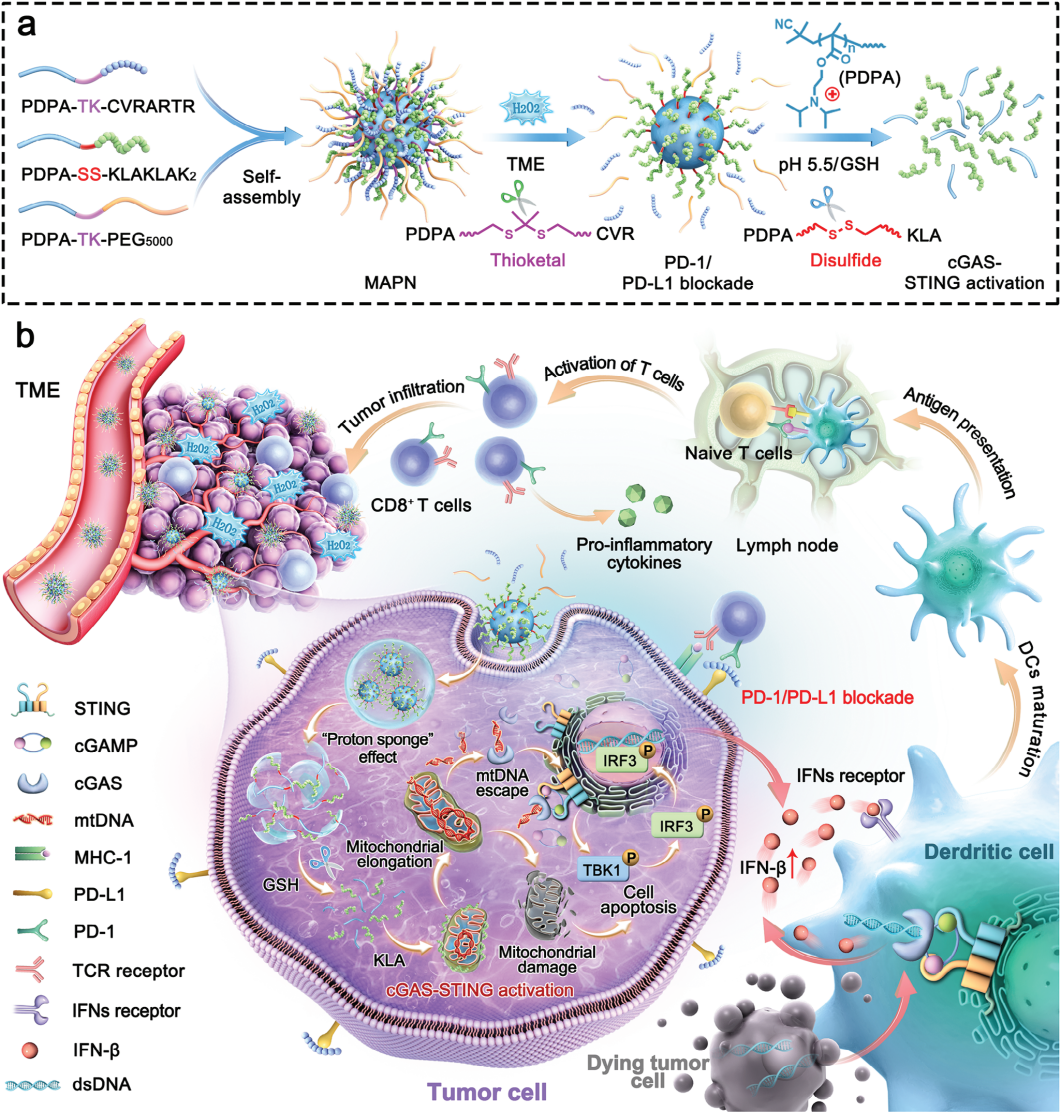
Schematic illustration for the design and immunotherapeutic mechanisms of MAPN. a) Preparation of MAPN and site‐specific release of CVR and KLA in response to high levels of H_2_O_2_ in tumor microenvironment and GSH‐riched intracellular environment, respectively. b) Schematic illustration of MAPN activating the cGAS‐STING pathway by promoting mtDNA leakage and blocking PD‐1/PD‐L1 pathway to initiate robust and durable anti‐tumor immune responses for cancer immunotherapy.

Although various nanocarriers have been utilized for in vivo delivery of therapeutic peptides, while carrier that can transport different peptides into specific action sites is rarely reported.^[^ ^14^ ^]^ Considering the high levels of hydrogen peroxide (H_2_O_2_) in TME and glutathione (GSH)‐riched intracellular environment,^[^ ^15^ ^]^ herein, we designed a multi‐stimuli activatable peptide nanodrug (MAPN) to precisely activate cGAS‐STING pathway and block PD‐1/PD‐L1 pathway through site‐specific delivery of AMPs and PD‐L1 antagonist peptides. The KLAKLAK_2_ (KLA) was screened out to act as a model AMP to promote mtDNA leakage by changing mitochondrial inner membrane permeability,^[^ ^10^, ^16^ ^]^ CVRARTR (CVR) was selected as the PD‐L1 antagonist peptide.^[^ ^12a^ ^]^ To achieve the site‐specific delivery in vivo, CVR and KLA were conjugated onto poly(2‐(diisopropyl amino) ethyl methacrylate) (PDPA) with a H_2_O_2_‐cleavable thioketal bond (TK) or GSH‐cleavable disulfide bond (SS) to obtain PDPA‐TK‐CVR and PDPA‐SS‐KLA, respectively. To improve circulation stability and protect peptides from enzymatic degradation, PDPA‐TK‐poly(ethylene glycol) (PDPA‐TK‐PEG_5000_) was further introduced to assemble with PDPA‐TK‐CVR and PDPA‐SS‐KLA to form MAPN (**Scheme** **1**a). With this structure, MAPN maintains stability during blood circulation, while detaching PEG corona to achieve enhanced tumor accumulation and cellular internalization after entering tumor tissues.^[^ ^17^ ^]^ The CVR would be released from MAPN in response to high levels of H_2_O_2_ in TME, achieving PD‐1/PD‐L1 pathway blockade. After internalization by tumor cells, PDPA triggers the endosomal escape of payload through the proton sponge effect,^[^ ^18^ ^]^ and then releases KLA in a GSH‐riched intracellular environment, ultimately inducing mtDNA leakage to activate the cGAS‐STING pathway (Scheme 1b). Both in vitro and in vivo results demonstrated that MAPN can precisely activate the cGAS‐STING pathway and block the PD‐1/PD‐L1 pathway, and thus initiating robust and durable anti‐tumor immune responses to inhibit the growth, recurrence, and metastasis of malignant tumors. Collectively, this study first proposed peptide‐based STING agonists and developed a feasible peptide delivery platform for cancer treatment.

## 2 Results and Discussion

### 2.1 AMPs‐Mediated cGAS‐STING Pathway Activation

**Figure 1:**
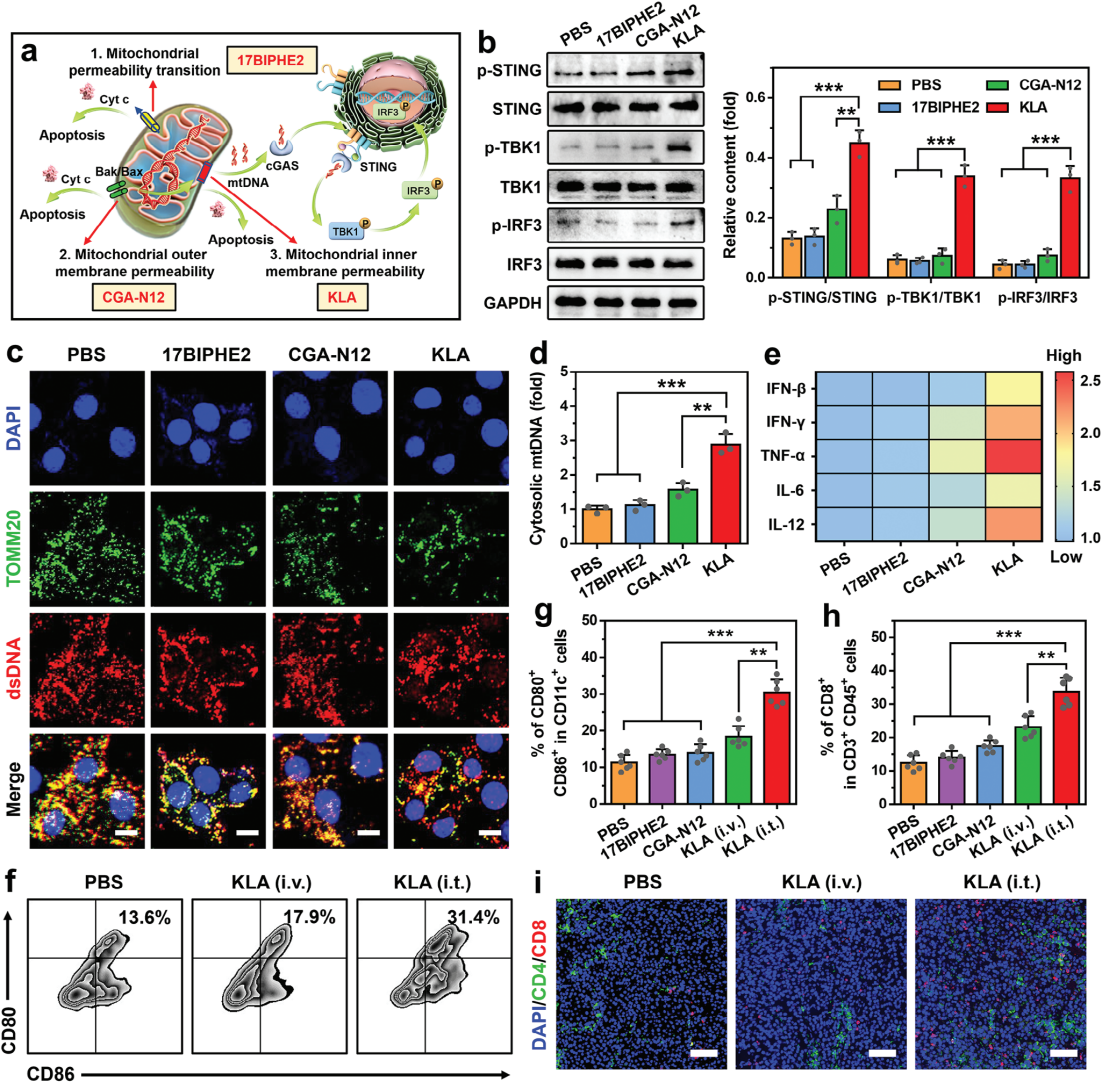
AMPs‐mediated cGAS‐STING pathway activation. a) Schematic illustration of three pathways for changing mitochondrial membrane permeability with different AMPs. b) Western bolt analysis of p‐STING, STING, p‐TBK1, TBK1, p‐IRF3, and IRF3 protein levels in B16F10 cells treated with different AMPs (n = 3). c) Immunofluorescence analysis of B16F10 cells treated with different AMPs. Mitochondria were stained with TOMM20 antibodies (green), dsDNA was stained with dsDNA Marker (red), and nuclei were stained with 4,6‐diamidino‐2‐phenylindole (DAPI, blue). Scale bars are 10 µm. d) The qRT‐PCR analysis of cytosolic mtDNA levels in B16F10 cells treated with different AMPs (n = 3). e) The secretion levels of various pro‐inflammatory cytokines in B16F10 cells treated with different AMPs (n = 6). f,g) Representative flow cytometric plots (f) and quantitative analysis (g) of DCs maturation (gated on CD80^+^ CD86^+^ CD11c^+^ cells) in lymph nodes (LNs) after different treatments (n = 6). h) Flow cytometric analysis of CD8^+^ CTLs infiltration (gated on CD45^+^ CD3^+^ CD8^+^ cells) in tumor tissues after different treatments (n = 6). i) Immunofluorescence analysis of CD4^+^ (green) and CD8^+^ CTLs (red) infiltration in tumor tissues after different treatments. Nuclei were stained with DAPI (blue), and Scale bars were 100 µm. Data are mean ± standard deviation (SD), Statistical significances were calculated via the Student's t‐test (^**^p < 0.01, ^***^p < 0.001).

We first examined three AMPs (detailed sequences in Table S1, Supporting Information) that change mitochondrial membrane permeability through three pathways to identify whether AMPs can activate the cGAS‐STING pathway in tumor cells (**Figure** **1**a).^[^ ^10a^ ^]^ As shown in Figure 1b, only KLA treatment (40 µm) remarkably increased the phosphorylation levels of STING, TBK1, and IRF3 proteins, indicating the effective cGAS‐STING pathway activation through changing mitochondrial inner membrane permeability. However, the other two AMPs that change mitochondrial permeability transition (17BIPHE2, 50 µm) or mitochondrial outer membrane permeability (CGA‐N12, 50 µm) slightly activated the cGAS‐STING pathway. To investigate the mechanism of AMPs‐mediated cGAS‐STING pathway activation, mtDNA and mitochondria were stained with dsDNA Marker and anti‐TOMM20 antibodies, respectively. Confocal laser scanning microscope (CLSM) images showed that green (mitochondria) and red fluorescence (dsDNA) almost overlapped in the cells treated with 17BIPHE2 and CGA‐N12 (Figure 1c), indicating that mtDNA was mainly located within mitochondria. In contrast, less cytosolic DNA (red) overlapped with mitochondria (green) in KLA‐treated cells (Pearson correlation coefficient = 0.464, Figure S1a, Supporting Information), indicating the leakage of mtDNA. The quantitative real‐time polymerase chain reaction (qRT‐PCR) analysis revealed 2.41‐fold and 1.87‐fold higher levels of cytosolic mtDNA in KLA‐treated cells than that in 17BIPHE2 and CGA‐N12‐treated cells, respectively (Figure 1d). Furthermore, we observed significantly higher levels of IFN‐β (2.03‐fold) and cGAMP (1.85‐fold) in KLA‐treated cells than that in PBS‐treated cells, confirming the effective activation of cGAS‐STING pathway after KLA treatment (Figure S1b,c, Supporting Information).

Next, the B16F10 tumor‐bearing C57BL/6 mouse model was established to investigate whether KLA can initiate efficient anti‐tumor immune responses by activating the cGAS‐STING pathway. The KLA was administrated either through intratumoral (i.t.) or intravenous (i.v.) injection at the dose of 7.5 mg kg^−1^, 17BIPHE2 and CGA‐N12 were employed as comparative groups. All experimental protocols were conducted within Anhui Medical University guidelines for animal research and were approved by the Institutional Animal Care and Use Committee (LLSC20230877). Flow cytometric analysis indicated that i.t. injection of KLA elicited 2.53‐fold higher DCs maturation and 2.36‐fold higher tumor‐infiltrating CD8^+^ T cells than that of PBS treatment, respectively (Figure 1f–h; Figure S2, Supporting Information). The enhanced tumor‐infiltrating CD8^+^ T cells after i.t. injection of KLA was also studied using CLSM (Figure 1i), demonstrating the effective activation of T cell anti‐tumor immunity. Similarly, significantly higher levels of pro‐inflammatory cytokines, such as IFN‐β, IFN‐γ, TNF‐α, IL‐6, and IL‐12, were observed in tumor tissues after i.t. injection of KLA compared with other comparative groups (Figure 1e). In contrast, i.v. injection of KLA displayed a weak effect on initiating systemic anti‐tumor immune responses, which was mainly due to quick degradation during blood circulation and undesirable tumor accumulation of KLA.^[^ ^13b^ ^]^ These results demonstrated that KLA can effectively activate the cGAS‐STING pathway through promoting mtDNA leakage, ultimately initiating robust anti‐tumor immune responses.

### 2.2 Preparation and Characterization of MAPN

**Figure 2:**
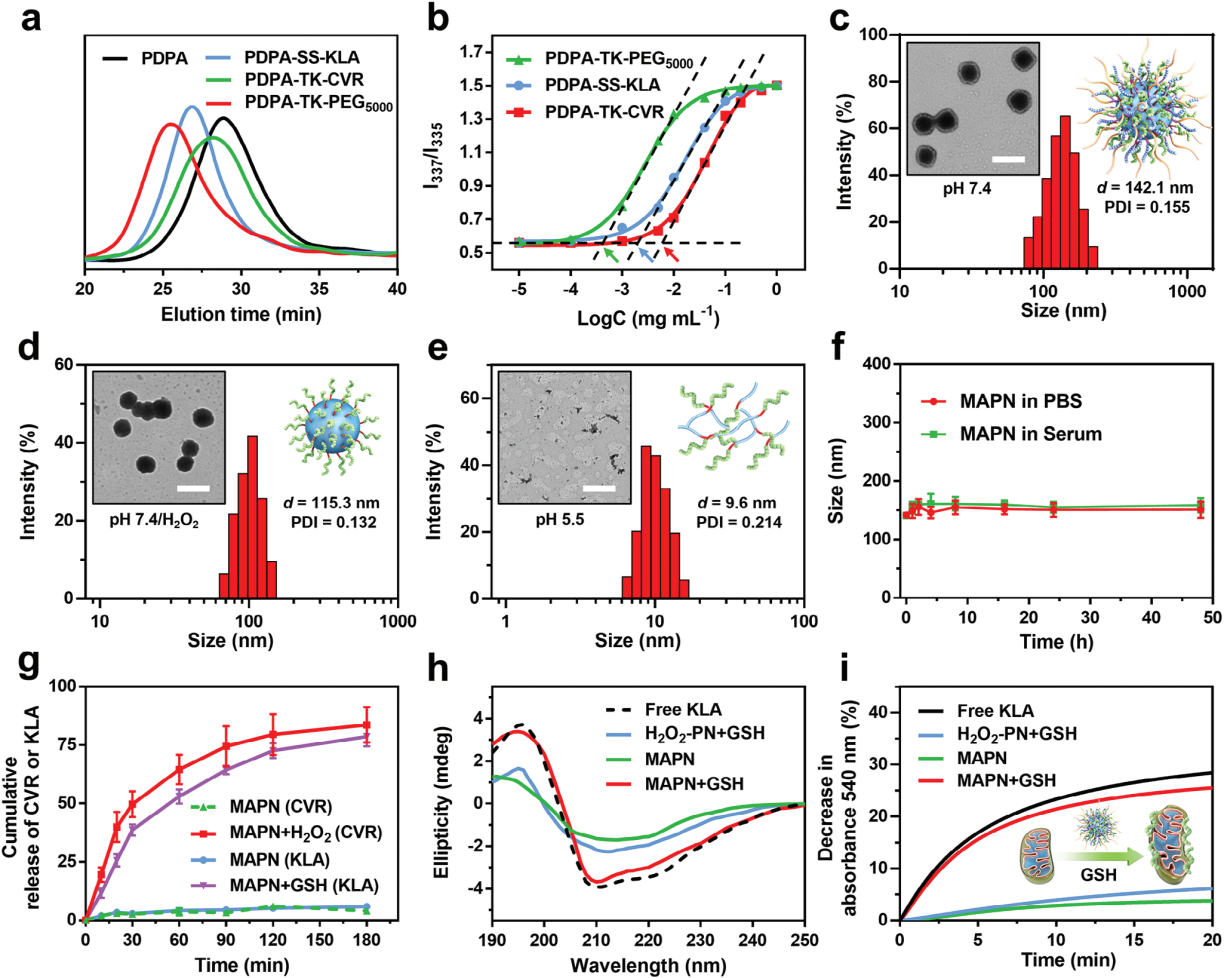
Synthesis and characterization of MAPN. a) GPC analysis of free PDPA, PDPA‐TK‐PEG_5000_, PDPA‐TK‐CVR, and PDPA‐SS‐KLA. b) Fluorescence intensity ratio of I_337_/I_335_ from pyrene excitation spectra against polymer concentrations. The arrows indicate the CMC values. c) TEM and DLS measurements of MAPN at pH 7.4. Scale bar is 200 nm. d) TEM and DLS measurements of MAPN at pH 7.4/H_2_O_2_. Scale bar is 200 nm. e) TEM and DLS measurement of MAPN at pH 5.5. Scale bar is 200 nm. f) Size changes of MAPN in PBS and mouse serum within 48 h (n = 3). g) Cumulative release of CVR and KLA from MAPN after incubation with H_2_O_2_ (100 µM) or GSH (10 mm), respectively (n = 3). h) Circular dichroism (CD) spectra of free KLA, H_2_O_2_‐PN, and MAPN with or without GSH. i) Decrease in absorbance at 540 nm of isolated mitochondria after incubation with free KLA, H_2_O_2_‐PN, and MAPN with or without GSH. Data are mean ± standard deviation (SD).

To effectively activate the cGAS‐STING pathway in vivo and initiate robust anti‐tumor immune responses, we designed a MAPN to precisely deliver PD‐L1 antagonist peptide (CVR) and KLA into TME and tumor cells, respectively. This MAPN was constructed by self‐assemble of three stimuli‐responsive polymers at a molar ratio of 1:1:1. The polymers were synthesized via atom‐transfer radical polymerization,^[^ ^19^ ^]^ followed by reacting with PEG_5000_, KLA, and CVR with a cleavable linker (TK or SS bond) to achieve PDPA‐TK‐PEG_5000_, PDPA‐TK‐CVR, and PDPA‐SS‐KLA, respectively (Figure S3, Supporting Information). The successful synthesis of these polymers was confirmed using ^1^H NMR and gel permeation chromatography (GPC) analysis (**Figure** **2**a; Figures S4–S6, Supporting Information). Thereafter, we measured the critical micelle concentrations (CMC) of polymers using pyrene probe.^[^ ^20^ ^]^ As shown in Figure 2b, the CMC values of PDPA‐TK‐PEG_5000_, PDPA‐TK‐KLA, and PDPA‐SS‐CVR are 4.8 × 10^−4^, 15.3 × 10^−4^ mg mL^−1^, and 41.7 × 10^−4^ mg mL^−1^, respectively, indicating the excellent capability to self‐assemble into stable micelle in aqueous solution. Transmission electron microscope (TEM) and dynamic light scattering (DLS) measurements revealed that the average particle size of MAPN was 142.1 nm (PDI: 0.155) with a spherical morphology at pH 7.4 (Figure 2c). After the addition of H_2_O_2_ (100 µm), a significant decrease in particle size from 142.1 to 106.3 nm (PDI: 0.132) was observed, suggesting the detachment of PEG corona in response to H_2_O_2_ (Figure 2d). Furthermore, no obvious micelle was observed by tuning the pH from 7.4 to 5.5, which was mainly due to micelle disintegration caused by the protonation of PDPA (Figure 2e).^[^ ^18^, ^20^ ^]^ Benefiting from shielding effects of PEG corona and near neutral zeta potential (5.1 ± 1.8 mV, Figure S7a, Supporting Information), MAPN exhibited excellent stability in mouse serum and PBS (Figure 2f), indicating its potential to avoid immune clearance in blood circulation. For better demonstration, H_2_O_2_‐responsive but GSH‐nonresponsive peptide nanodrug (H_2_O_2_‐PN) and GSH‐responsive but H_2_O_2_‐nonresponsive peptide nanodrug (GSH‐PN) were prepared with similar methods and used for follow‐up studies (detailed synthesis and characterization in Figures S8–S10, Supporting Information).

We then evaluate the site‐specific release of CVR and KLA in response to H_2_O_2_ and GSH, respectively. As shown in Figure 2g and Figure S7b (Supporting Information), > 80% CVR was released from MAPN within 180 min in the presence of H_2_O_2_ (100 µm), while little CVR was released from GSH‐PN and MAPN in the absence of H_2_O_2_, demonstrating the H_2_O_2_‐responsive release of CVR. Similarly, only a significant release of KLA (77.7%) was observed from MAPN in the presence of GSH (10 mm), suggesting the GSH‐responsive release of KLA. To investigate the potential of MAPN in damaging mitochondria membranes, a trifluoroethanol (TFE)/PBS (1:9, v:v) mixed solution was used to induce the conformation change of KLA.^[^ ^21^ ^]^ As shown in Figure 2h, H_2_O_2_‐PN+GSH and MAPN displayed weak helical structures, which was mainly due to less mobility of KLA in the form of the micelle. In contrast, MAPN+GSH showed similar conformation to free KLA, indicating its potential to damage the mitochondria membrane in a GSH‐riched intracellular environment. The morphological changes of isolated mitochondria were analyzed by monitoring the absorbance at 540 nm to confirm the mitochondrial membrane damage ability of MAPN.^[^ ^13b^ ^]^ As shown in Figure 2i, a significant decrease in absorbance at 540 nm was observed in isolated mitochondria treated with MAPN+GSH, indicating the effective mitochondrial membrane damage.

### 2.3 PD‐1/PD‐L1 Pathway Blockade, Cellular Internalization, Endosomal Escape, and In Vivo Tumor Accumulation of MAPN

**Figure 3:**
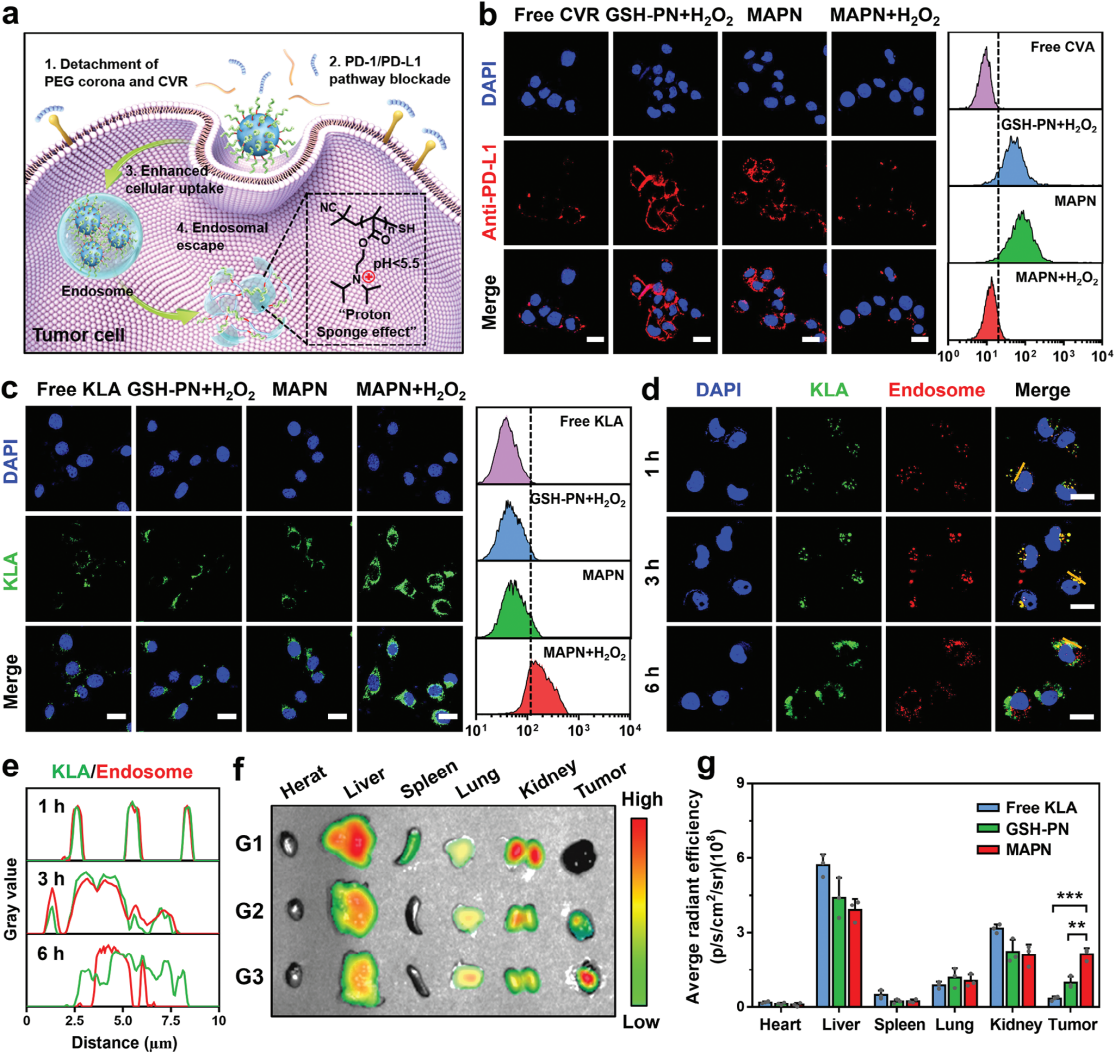
In vitro PD‐1/PD‐L1 pathway blockade, cellular internalization, endosomal escape, and in vivo tumor accumulation of MAPN. a) Schematic illustration for PD‐1/PD‐L1 pathway blockade, cellular internalization, and endosomal escape of MAPN. b) CLSM and flow cytometric analysis of anti‐PD‐L1 antibody‐incubated B16F10 cells after pre‐treated with free CVR, GSH‐PN, and MAPN under different conditions for 2 h at 37 °C. Anti‐PD‐L1 was labeled with Alexa Fluor 594 (red). Nuclei were counterstained with DAPI (blue). Scale bars are 20 µm. c) CLSM and flow cytometric measurement of B16F10 cells after incubated with free KLA, GSH‐PN, and MAPN under different conditions for 4 h at 37 °C. KLA was labeled with FITC (green). Nuclei were counterstained with DAPI (blue). Scale bars are 20 µm. d) CLSM images of B16F10 cells treated with MAPN+H_2_O_2_ for 1 h, 3 h, and 6 h, respectively. KLA was labeled with FITC (green). Endosomes were stained with a LysoTracker probe (red). Nuclei were stained with DAPI (blue). Scale bars are 20 µm. e) The normalized intensity profile of ROIs across the yellow lines in (d). f) Ex vivo fluorescence images of major organs and tumors in mice after intravenous injection of free KLA (G1), GSH‐PN (G2), and MAPN (G3) at 24 h post‐injection. KLA was labeled with Cy5.5. g) Fluorescence intensity analysis based on *ex vivo* fluorescence images (n = 3). Data are presented as mean ± standard deviation (SD). Statistical significance in (g) was calculated via the Student's t‐test (^**^p < 0.01, ^***^p < 0.001).

The H_2_O_2_‐responsive release of CVR and detachment of PEG corona would block the PD‐1/PD‐L1 pathway and enhance the cellular internalization of KLA by tumor cells (**Figure** **3**a). To demonstrate the MAPN‐mediated PD‐1/PD‐L1 pathway blockade, we first studied the PD‐1/PD‐L1 pathway blockade at various concentrations of free CVR. As shown in Figure S11a (Supporting Information), CVR effectively blocked the PD‐1/PD‐L1 pathway when the concentration is greater than 4 µm. Next, B16F10 cells were treated with MAPN+H_2_O_2_ and further incubated with Alexa Fluor 594‐labeled (red) anti‐PD‐L1 antibodies, PD‐1/PD‐L1 pathway blockade was analyzed using CLSM and flow cytometry. Compared with the cells treated with MAPN in the absence of H_2_O_2_, much weaker fluorescence intensity (red) was observed in the surface of cells treated with MAPN+H_2_O_2_ (0.21‐fold over that of MAPN), indicating the effective blockade of PD‐1/PD‐L1 pathway (Figure 3b; Figure S11b, Supporting Information). To evaluate the cellular internalization of KLA, MAPN was prepared with FITC‐labeled KLA and incubated with B16F10 cells for CLSM analysis, and GSH‐PN was set as a comparative group. As shown in Figure 3c, 4.51‐fold, 3.75‐fold, and 2.87‐fold higher fluorescence intensity were observed in the cells treated with MAPN+H_2_O_2_ than that in the cells treated with free KLA, MAPN, and GSH‐PN+H_2_O_2_, indicating the enhanced cellular uptake of KLA by MAPN in the presence of H_2_O_2_. Further analysis with flow cytometry also confirmed these results (Figure 3c; Figure S11c, Supporting Information), which could be attributed to the detachment of PEG corona and increased zeta potential (Figure S7b, Supporting Information). After entering tumor cells, the protonation of PDPA in an acidic endosome environment (pH 4.5–5.5) promotes the endosomal escape of payload through the proton sponge effect. To demonstrate, B16F10 cells were incubated with MAPN in the presence of H_2_O_2_ and directly observed using CLSM at different time points. Endosomes were stained with LysoTracker probe (red). As shown in Figure 3d,e, and Figure S11d (Supporting Information), KLA (green) and endosomes (red) almost overlapped at 1 and 3 h post‐incubation. In contrast, the overlap of green and red fluorescence markedly reduced after 6 h incubation (Pearson correlation coefficient = 0.318), indicating the successful endosomal escape of payload. As described above, benefiting from the PEG corona and neutral zeta potential under a normal physiological environment, MAPN can maintain stability in serum (Figure 2f), which helps to prolong blood circulation.^[^ ^15^, ^22^ ^]^ To demonstrate it, free KLA, GSH‐PN, and MAPN containing Cy5.5‐labeled KLA were injected into C57BL/6 mice via tail vein, respectively. As shown in Figure S12a (Supporting Information), MAPN exhibited significantly longer in vivo half‐time than free KLA, suggesting the potential of MAPN in avoiding rapid immune clearance and prolonging blood circulation. The in vivo biodistribution of free KLA, GSH‐PN, and MAPN were then investigated on a B16F10 tumor‐bearing C57BL/6 model and analyzed using in vivo imaging system (IVIS). As shown in Figure 3f,g, and Figure S12b (Supporting Information), significantly higher fluorescence intensity (2.3‐ and 7.3‐fold over that of GSH‐PN and free KLA, respectively) were observed from tumors in MAPN‐treated mice than that of other groups. Due to the comparable size and zeta potential between GSH‐PN and MAPN (Table S2, Supporting Information), the enhanced tumor accumulation of MAPN could be attributed to the H_2_O_2_‐responsive detachment of PEG corona, which in turn expose the core with a higher zeta potential and subsequently promote the cellular internalization.^[^ ^15^, ^17^ ^]^ In general, MAPN maintains a stable structure during blood circulation, while releasing CVR to block the PD‐1/PD‐L1 pathway after entering tumor tissues. Additionally, the detachment of PEG corona in TME can enhance the tumor accumulation and cellular internalization of MAPN, followed by the protonation of PDPA triggering endosomal escape and release of KLA into the cytoplasm through the proton sponge effect.

### 2.4 MAPN Promotes mtDNA Leakage to Activate cGAS‐STING Pathway

**Figure 4:**
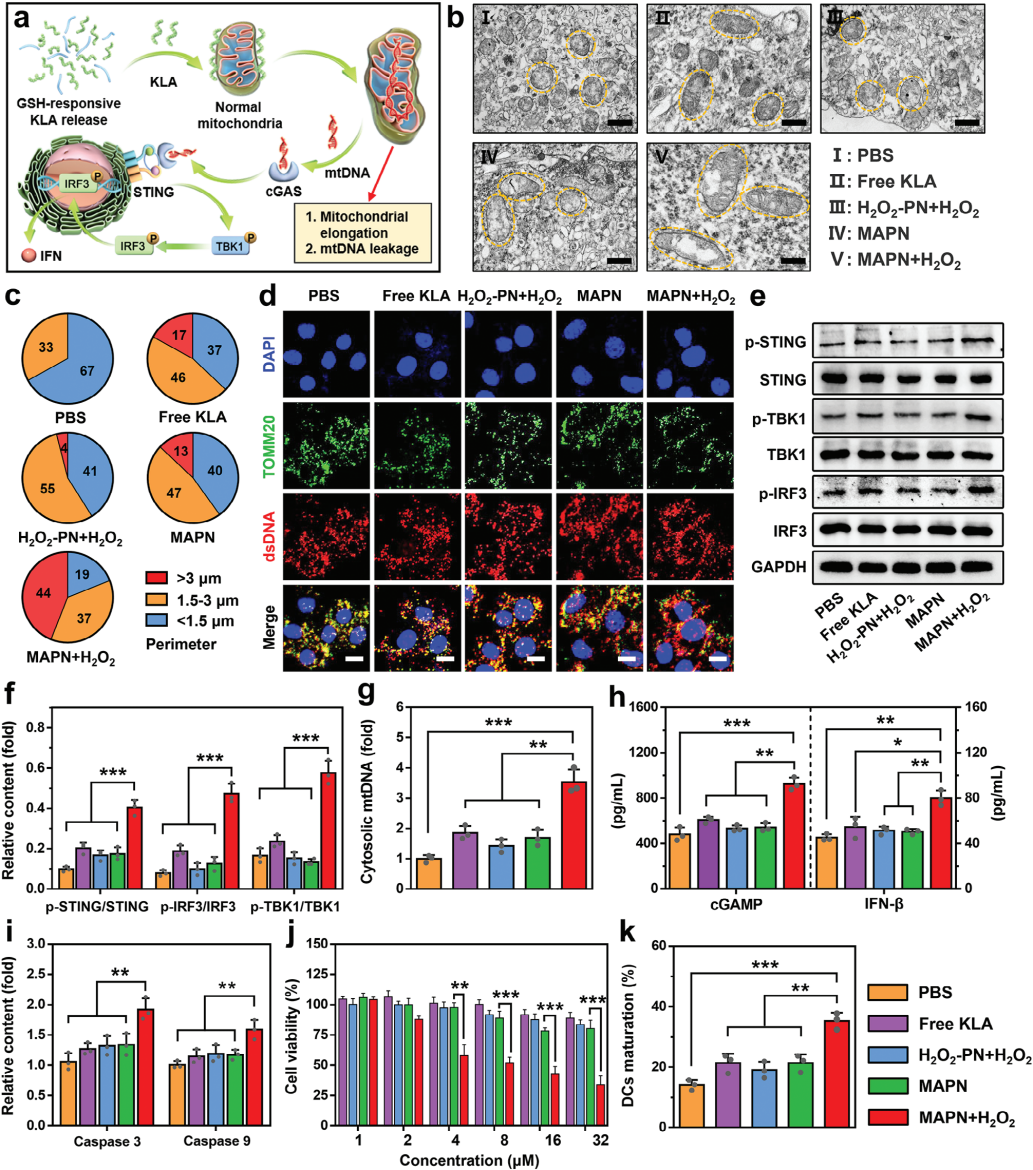
MAPN‐mediated activation of cGAS‐STING pathway. a) Schematic illustration of MAPN‐mediated mitochondrial elongation, mtDNA leakage, and activation of cGAS‐STING pathway. b) Bio‐TEM analysis of mitochondrial morphologies of B16F10 cells after different treatments. Scale bars are 0.5 µm. c) Mitochondrial perimeter analysis of bio‐TEM images. d) Immunofluorescence analysis of B16F10 cells after different treatments. Nuclei were stained with DAPI (blue), mitochondria were stained with TOMM20 antibodies (green), and dsDNA was stained with a dsDNA Marker (red). Scale bars are 10 µm. e,f) Western bolt images (e) and quantitative analysis (f) of p‐STING, STING, p‐TBK1, TBK1, p‐IRF3, and IRF3 protein levels in B16F10 cells after different treatments (n = 3). g) The qRT‐PCR analysis of cytosolic mtDNA levels in B16F10 cells after different treatments (n = 3). h) Relative secretion levels of cGAMP and IFN‐β in B16F10 cells after different treatments (n = 3). i) Relative expression levels of caspase‐3 and caspase‐9 in B16F10 cells after different treatments (n = 3). j) The viability of B16F10 cells treated with different formations at various concentrations (n = 3). k) Flow cytometric analysis of matured BMDCs (gated on CD11c^+^ CD80^+^ CD86^+^ cells) after different treatments (n = 3). Data are presented as mean ± standard deviation (SD). Statistical significance was calculated via the Student's t‐test (^*^p < 0.05, ^**^p < 0.01, ^***^p < 0.001).

The PDPA‐SS‐KLA that successfully escaped from endosomes could be cleaved in a GSH‐riched intracellular environment, subsequently releasing mtDNA by destroying mitochondrial membranes, ultimately activating the cGAS‐STING pathway (**Figure** **4**a). The destruction of the mitochondrial membrane is mainly characterized by mitochondrial elongation and mtDNA leakage.^[^ ^23^ ^]^ Bio‐TEM was first employed to directly observe mitochondrial morphology. As shown in Figure 4b,c, obvious mitochondrial elongation was observed in cells treated with MAPN+H_2_O_2_, the proportion of mitochondria with a perimeter exceeding 3 µm increased from 0% to 44% compared with PBS treatment. In contrast, H_2_O_2_‐PN+H_2_O_2_ hardly changed mitochondrial morphology, which may be ascribed to less flexibility and mobility of KLA after conjugation with PDPA. Furthermore, no significant mitochondrial morphological change was observed in cells treated with free KLA and MAPN due to insufficient cellular internalization. Next, mtDNA and mitochondria were stained with dsDNA Marker (red) and anti‐TOMM20 antibodies (green) to investigate the mtDNA leakage. As shown in Figure 4d, green and red fluorescence were well colocalized in the cells treated with PBS, free KLA, and H_2_O_2_‐PN+H_2_O_2_, indicating that mtDNA was mainly located within mitochondria. In contrast, less mtDNA was overlapped with mitochondria in the cells treated with MAPN+H_2_O_2_ (Pearson correlation coefficient = 0.357), indicating the mtDNA leakage from mitochondria. Further analysis with qRT‐PCR further confirmed the leakage of mtDNA after MAPN+H_2_O_2_ treatment, and the level of cytosolic DNA was 3.62‐fold higher than that of PBS treatment (Figure 4g). These results demonstrated that MAPN can effectively promote the leakage of mtDNA in TME. To investigate whether MAPN‐mediated mtDNA leakage can activate the cGAS‐STING pathway, the phosphorylation levels of STING, TBK1, and IRF3 proteins were examined with the western bolt. Notably, compared with other groups, MAPN+H_2_O_2_ treatment significantly elevated the phosphorylation levels of STING, TBK1, and IRF3, demonstrating the effective activation of the cGAS‐STING pathway (Figure 4e,f). The secretion levels of cGAMP and IFN‐β, which are important markers of cGAS‐STING pathway activation, increased to 1.81‐fold and 1.75‐fold higher than those of the PBS group, respectively (Figure 4h). In addition to activating the cGAS‐STING pathway, KLA can also kill the cells through a rapid and non‐receptor‐mediated membranolytic mechanism.^[^ ^10a^ ^]^ As shown in Figure 4i, MAPN markedly increased the activity of caspase‐3 and caspase‐9 in the presence of H_2_O_2_, indicating its excellent membranolytic ability. We also found that MAPN+H_2_O_2_ exhibited much higher cytotoxicity than free KLA at various concentrations (Figure 4j). Such toxicity enhancement is consistent with our previous results, which could be attributed to enhanced cellular internalization and efficient endosomal escape of KLA by MAPN. In addition, MAPN‐mediated DCs maturation was explored using a transwell experiment (Figure S13a, Supporting Information). The Bone marrow‐derived dendritic cells (BMDCs) were extracted from C57BL/6 mice and incubated with B16F10 cells pretreated with different formations. As shown in Figure 4k and Figure S13b (Supporting Information), compared to the PBS group, the maturation ratio of BMDCs (gated on CD11c^+^ CD80^+^ CD86^+^ cells) significantly increased from 14.2 to 35.8% after co‐cultured with MAPN+H_2_O_2_‐pretreated B16F10 cells, which may be attributed to secreted IFN‐β and tumor‐derived cytosolic dsDNA.^[^ ^24^ ^]^ These results indicated that MAPN can effectively promote mtDNA leakage and cell apoptosis through the membranolytic mechanism, ultimately activating the cGAS‐STING pathway.

### 2.5 In Vivo Tumor Inhibition and T Cell Immunity Activation with MAPN Treatment

**Figure 5:**
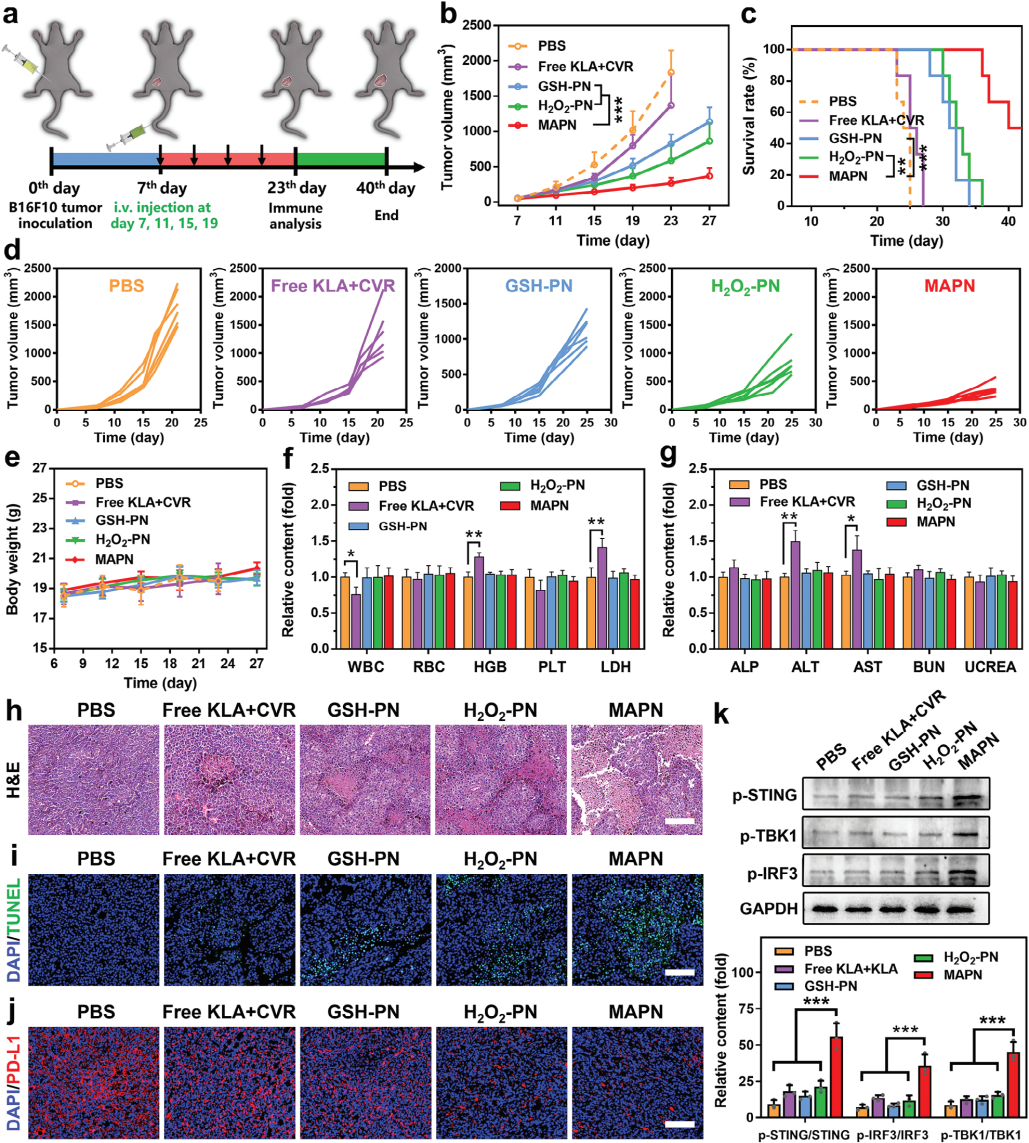
In vivo tumor inhibition with MAPN treatment. a) Illustration of treatment schedule to evaluate the anti‐tumor activity of MAPN. b) Tumor growth curves of B16F10 tumor‐bearing mice after different treatments (n = 6). c) Survival curves of of B16F10 tumor‐bearing mice after different treatments (n = 6). d) Individual tumor growth curves of B16F10 tumor‐bearing mice after different treatments (n = 6). e) Body weight changes of B16F10 tumor‐bearing mice after different treatments (n = 6). f) The relative concentrations of white blood cell (WBC), red blood cell (RBC), hemoglobin (HGB), platelets (PLT), and lactate dehydrogenase (LDH) in the blood of tumor‐bearing mice after different treatments (n = 3). g) The relative concentrations of alkaline phosphatase (ALP), alanine transaminase (ALT), aspartate transaminase (AST), blood urea nitrogen (BUN), and urea creatinine (UCREA) in the blood of tumor‐bearing mice after different treatments (n = 3). h,i) H&E (h) and TUNEL (i) staining of tumor sections from mice after different treatments. Scale bars are 100 µm. j) Immunofluorescence analysis of PD‐L1 in tumors from mice after different treatments. PD‐L1 were stained with Alex Fluor 594 (red) labeled anti‐PD‐L1 antibodies. Nuclei were counterstained with DAPI (blue). Scale bars are 100 µm. k) Western bolt analysis of p‐STING, p‐TBK1, and p‐IRF3 protein levels in tumors from mice after different treatments (n = 3). Data are presented as mean ± standard deviation (SD). Statistical significance in (b), (c), and (e) were calculated via the one‐way ANOVA with Tukey's post hoc test (^**^p < 0.01, ^***^p < 0.001). Statistical significance in (f), (g), and (k) were calculated via the Student's t‐test (^*^p < 0.05, ^**^p < 0.01, ^***^p < 0.001).

Encouraged by the effective cGAS‐STING pathway activation and PD‐1/PD‐L1 pathway blockade in vitro, we further investigated the in vivo anti‐tumor effects of MAPN on a B16F10 tumor‐bearing model (**Figure** **5**a). MAPN was intravenously injected into tumor‐bearing mice every four days for four times, free KLA+CVR, GSH‐PN, and H_2_O_2_‐PN were set as comparative groups. As shown in Figure 5b,d, MAPN treatment significantly inhibited the growth of malignant tumors with a tumor growth inhibition rate of 78%, which was obviously higher than single ICB (H_2_O_2_‐PN) and cGAS‐STING pathway activation (GSH‐PN). Moreover, no obvious tumor inhibition was observed in mice treated with free KLA+CVR, which may be attributed to the rapid degradation of peptides in vivo. Survival curves of mice showed the median survival of B16F10 tumor‐bearing mice received MAPN treatment was extended to 40 days, which was significantly longer than that of other comparative groups (Figure 5c). Subsequently, hematoxylin‐eosin (H&E) and terminal deoxynucleotidyl transferase dUTP nick end labeling (TUNEL) staining further confirmed the excellent anti‐tumor effects of MAPN (Figure 5h,i). More importantly, no obvious weight loss was observed in the mice after MAPN treatment, indicating the excellent biocompatibility of MAPN. After one week of treatment, some mice were sacrificed, and blood samples and major organs were collected for histopathological, blood biochemical, and blood routine analysis. As shown in Figure 5f,g, and Figure S14 (Supporting Information), obvious variations in white blood cell (WBC), hemoglobin (HGB), lactate dehydrogenase (LDH), alanine transaminase (ALT), and aspartate transaminase (AST) levels were observed in the mice treated with free KLA+CVR, which was mainly due to hemolysis caused by cationic peptides.^[^ ^25^ ^]^ In contrast, no notable change in these tests was observed from MAPN‐treated mice, further demonstrating the biosafety of MAPN treatment.

**Figure 6:**
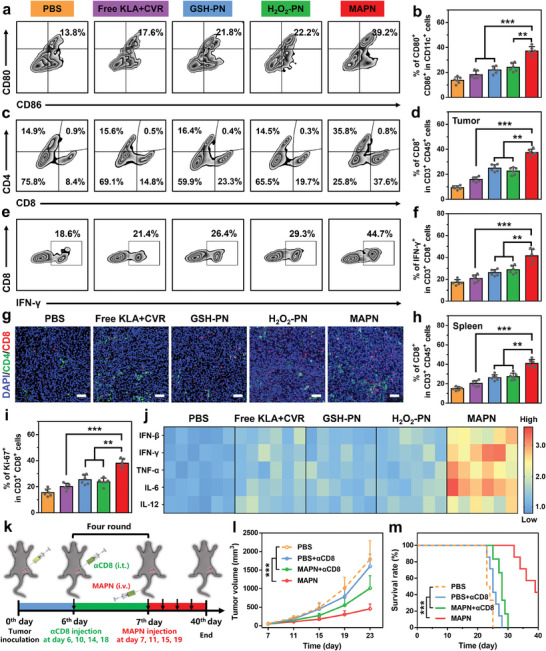
Activation of T‐cell anti‐tumor immunity with MAPN treatment. a,b) Representative flow cytometric plots (a) and quantitative analysis (b) of DCs maturation in LNs after different treatments (n = 6). c,d) Representative flow cytometric plots (c) and quantitative analysis (d) of CD8^+^ CTLs infiltration (gated on CD45^+^ CD3^+^ CD8^+^ cells) in tumor tissues after different treatments (n = 6). e,f) Representative flow cytometric plots (e) and quantitative analysis (f) of IFN‐γ^+^ CD8^+^ CTLs in tumor tissues after different treatments (n = 6). g) Immunofluorescence analysis of CD4^+^ (green) and CD8^+^ (red) CTLs infiltration in tumor tissues after different treatments. Nuclei were stained with DAPI (blue). Scale bars were 100 µm. h) Flow cytometric quantitative analysis of CD8^+^ CTLs population in spleens after different treatments (n = 6). i) Flow cytometric quantitative analysis of Ki67^+^ CD8^+^ CTLs in tumor tissues after different treatments (n = 6). j) Relative levels of proinflammatory cytokines (IFN‐β, IFN‐γ, TNF‐α, IL‐12, and IL‐6) in tumor tissues after different treatments (n = 6). k) Illustration of the treatment schedule to evaluate the anti‐tumor effects of MAPN in CD8^+^ T cell‐depleted B16F10 tumor‐bearing mice. l,m) Tumor growth curves (l) and survival curves (m) of CD8^+^ T cell‐depleted B16F10 tumor‐bearing mice after different treatments (n = 6). Data are presented as mean ± standard deviation (SD). Statistical significance in (b), (d), (f), (h), and (i) were calculated via the Student's t‐test (^*^p < 0.05, ^**^p < 0.01, ^***^p < 0.001). Statistical significance in (l) and (m) were calculated via the one‐way ANOVA with Tukey's post hoc test (^***^p < 0.001).

To elucidate the anti‐tumor mechanism of MAPN treatment, we first examined the PD‐1/PD‐L1 blockade and cGAS‐STING pathway activation in tumor tissues. Immunofluorescence assay revealed the effective PD‐1/PD‐L1 pathway blockade in tumors after MAPN treatment, which could be attributed to the release of CVR in response to TME (Figure 5j). Western bolt analysis showed a significant elevation in phosphorylation levels of STING, TBK1, and IRF3 proteins in tumors from the mice after MAPN treatment, demonstrating the effective activation of the cGAS‐STING pathway (Figure 5k; Figure S15, Supporting Information). With the PD‐1/PD‐L1 pathway blockade and cGAS‐STING pathway activation, MAPN would initiate robust anti‐tumor immune responses. As expected, compared to PBS treatment, MAPN increased the frequency of matured DCs (CD11c^+^ CD80^+^ CD86^+^ cells) in lymph nodes (LNs) from 13.8% to 39.2% (**Figure** **6**a,b), and facilitated the intratumoral infiltration of CD8^+^ CTLs from 8.4% to 39.6% (Figure 6c,d). The enhanced intratumoral infiltration of CD8^+^ CTLs was also demonstrated by immunofluorescence assay (Figure 6g). Furthermore, MAPN markedly elevated the frequencies of IFN‐γ^+^ (2.4‐fold over that of PBS group) and Ki67^+^ (2.2‐fold over that of PBS group) CD8^+^ CTLs in tumor tissues, and frequency of CD8^+^ T cells (2.3‐fold over that of PBS group) in spleens (Figure 6e,f,h,i,; Figure S16, Supporting Information), indicating the activation of systemic immune responses. Similar tendencies were also observed in the secretion levels of pro‐inflammatory cytokines in tumor tissues, including IFN‐β, IFN‐γ, TNF‐α, IL‐12, and IL‐6 (Figure 6j). To demonstrate the activation of T cell anti‐tumor immunity, we further studied the anti‐tumor effects of MAPN on a CD8^+^ T cell‐depleted B16F10 tumor‐bearing model by intratumoral injection of αCD8.^[^ ^17b^ ^]^ The CD8^+^ T cell‐depleted B16F10 tumor‐bearing mice treated with MAPN and PBS were denoted as “MAPN+αCD8” and “PBS+αCD8”, respectively (Figure 6k). As shown in Figure 6l,m, and Figure S17 (Supporting Information), MAPN treatment showed much weaker anti‐tumor effects in CD8^+^ T cell‐depleted B16F10 tumor‐bearing mice (MAPN+αCD8) than that in normal B16F10 tumor‐bearing mice, demonstrating the importance of T cell immunity in MAPN‐mediated anti‐tumor effects. Collectively, MAPN can synergistically activate the cGAS‐STING pathway and block the PD‐1/PD‐L1 pathway to initiate efficient T cell anti‐tumor immunity, and thus effectively inhibiting the growth of malignant tumors and prolonging the survival rates of tumor‐bearing mice.

### 2.6 The Long‐Term Immune Memory Effects with MAPN Treatment

**Figure 7:**
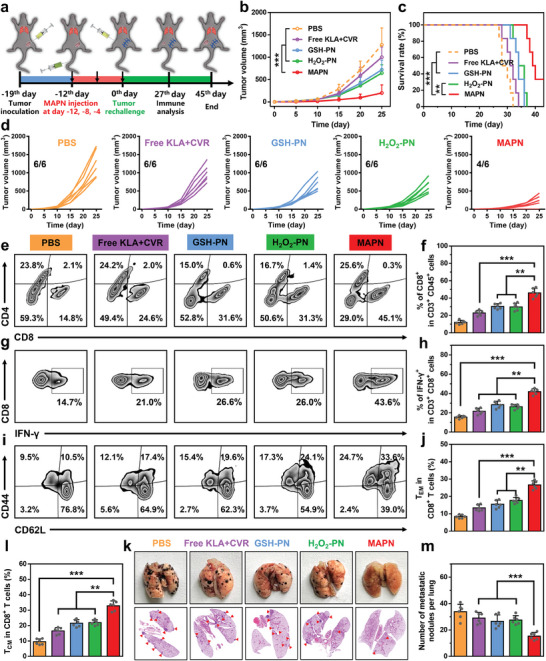
MAPN‐mediated immune memory effects to prevent the recurrence and metastasis of malignant tumors. a) Illustration of treatment schedule for tumor recurrence and metastasis in B16F10 tumor‐bearing mice. b,c) Rechallenged tumor growth curves (b) and survival curves (c) of the mice after different treatments (n = 6). d) Individual rechallenged tumor growth curves after different treatments. e,f) Representative flow cytometric plots (e) and quantitative analysis (f) of CD8^+^ CTLs infiltration in the rechallenged tumors after different treatments (n = 6). g,h) Representative flow cytometric plots (g) and quantitative analysis (h) of IFN‐γ^+^ CD8^+^ CTLs in the rechallenged tumors after different treatments (n = 6). i–l) Representative flow cytometric plots (i) and quantitative analysis of T_EM_ (gated on CD3^+^ CD8^+^ CD44^+^ CD62L^−^ cells) (j) and T_CM_ (gated on CD3^+^ CD8^+^ CD44^+^ CD62L^+^ cells) (l) in the spleens of the mice after different treatments (n = 6). k) Representative images and H&E staining analysis of lung sections in mice after different treatments. m) Quantitation of lung metastatic nodules in the mice after different treatments (n = 6). Data are presented as mean ± standard deviation (SD). Statistical significance in (f), (h), (j), (l), and (m) were calculated via the Student's t‐test (^**^p < 0.01, ^***^p < 0.001). Statistical significance in (b) and (c) were calculated via the one‐way ANOVA with Tukey's post hoc test (^**^p < 0.01, ^***^p < 0.001).

The activation of systemic anti‐tumor immune responses could trigger long‐term immune memory effects to prevent the recurrence and metastasis of malignant tumors.^[^ ^26^ ^]^ To explore the potential of MAPN in preventing tumor recurrence and metastasis, we constructed a rechallenged tumor model as indicated in **Figure** **7**a. After three treatments, primary tumors in the left flank were removed surgically at day 0 and then rechallenged with B16F10 cells in the right flank. As shown in Figure 7b–d, MAPN delayed the growth of rechallenged tumors with an inhibition rate of 82.3% and significantly improved the survival rates of tumor‐rechallenged mice. Further analysis with H&E and TUNEL staining revealed the most severe cell apoptosis in rechallenged tumors after pretreated with MAPN compared with other groups (Figure S18, Supporting Information), confirming the inhibitory effects on rechallenged tumors. Next, we investigated the activation of T cell anti‐tumor immunity in the rechallenged tumors. Compared with the PBS group, MAPN elevated the frequencies of tumor‐infiltrating CD8^+^ and IFN‐γ^+^ CD8^+^ CTLs to 3.1‐fold and 2.9‐fold in the rechallenged tumors, respectively (Figure 7e–h). Immunofluorescence and enzyme‐linked immunosorbent assays also demonstrated the efficient intratumoral infiltration of CD8^+^ CTLs and elevated levels of pro‐inflammatory cytokines in the rechallenged tumors (Figures S19,S20, Supporting Information). Furthermore, significantly higher fractions of effector memory T cells (T_EM_, 2.6‐fold over that of the PBS group) and central memory T cells (T_CM_, 3.2‐fold over that of the PBS group) were observed in spleens from the MAPN‐treated mice (Figure 7i–l), indicating the effective activation of long‐term immune memory effects. Eventually, lungs were collected to evaluate tumor metastasis after different treatments. As shown in Figure 7k,m, compared with other comparative groups, much less lung metastatic nodules were observed from the MAPN‐treated mice. Overall, these results demonstrated that MAPN can effectively prevent the recurrence and metastasis of malignant tumors by triggering long‐term immune memory effects.

## 3 Conclusion

In conclusion, we have proposed AMPs‐based STING agonists and demonstrated a multi‐stimuli activatable peptide nanodrug (MAPN) to precisely activate the cGAS‐STING pathway and block PD‐1/PD‐L1 pathway for effective cancer immunotherapy. To construct MAPN, AMP (KLA) and PD‐L1 antagonist peptide (CVR) were first conjugated onto PDPA with a disulfide bond or thioketal bond, and then self‐assembled with a detachable PEGylated polymer, which enables MAPN to avoid immune clearance during blood circulation and efficiently accumulate at tumor tissues. With this design, MAPN achieved the site‐specific release of KLA and CVR in response to the high levels of H_2_O_2_ in TME and intracellular GSH, activating the cGAS‐STING pathway and blocking the PD‐1/PD‐L1 pathway, ultimately initiating robust and durable T cell anti‐tumor immunity to inhibit the growth, recurrence, and metastasis of malignant tumors. In view of the drawbacks of existing small molecule STING agonists, such as low bioavailability, nonspecificity, and adverse effects, promoting mtDNA leakage with AMPs may be a promising strategy for activating cGAS‐STING pathway, and giving a new insight for the design of novel STING agonists. Furthermore, MAPN presents a universal delivery platform for the effective synergy of different peptides.

## Conflict of Interest

The authors declare no conflict of interest.

## Supporting information

Supporting Information

## Data Availability

The data that support the findings of this study are available from the corresponding author upon reasonable request.
