# Supplementary material for: Precisely Activating cGAS‐STING Pathway with a Novel Peptide‐Based Nanoagonist to Potentiate Immune Checkpoint Blockade Cancer Immunotherapy
Source: Adv Sci (Weinh). 2024 Jan 17;11(15):2309583. doi: 10.1002/advs.202309583 (PMC11022698; doi:10.1002/advs.202309583)
Supplement: Supplementary file 1 — Supporting Information [file ADVS-11-2309583-s001.pdf]

ADVANCED SCIENCE
Open Access

Supporting Information

for *Adv. Sci.*, DOI 10.1002/advs.202309583

Precisely Activating cGAS-STING Pathway with a Novel Peptide-Based Nanoagonist to Potentiate Immune Checkpoint Blockade Cancer Immunotherapy

Yumeng Xing, Ao Peng, Jianhui Yang, Zhifei Cheng, Yi Yue, Feilong Liu, Fenghe Li, Yang Liu and Qi Liu**

Supporting Information

Precisely Activating cGAS-STING Pathway with a Novel Peptide-based Nanoagonist to Potentiate Immune Checkpoint Blockade Cancer Immunotherapy

Yumeng Xing, Ao Peng, Jianhui Yang, Zhifei Cheng, Yi Yue, Feilong Liu, Fenghe, Li, Yang Liu, and Qi Liu**

Y. Xing, A. Peng, J. Yang, Y. Yue, Dr. F. Liu, Dr. F. Li, Prof. Q. Liu
School of Pharmacy, Anhui Medical University
Hefei 230032, China
E-mail: liuqi@ahmu.edu.cn

Prof. Y. Liu
College of Chemistry, Nankai University
Tianjin 300071, China
E-mail: yliu@nankai.edu.cn

Y. Xing
College of Chemistry and Chemical Engineering, Xiamen University
Xiamen 361005, China

Dr. Z. Cheng
School of Pharmacy, Anhui University of Chinese Medicine
Hefei 230012, China

Keywords: multi-stimuli activatable, cGAS-STING activation, antimicrobial peptides, PD-1/PD-L1 blockade, cancer immunotherapy

Table of Contents

Materials..3

Instrucments..3

Synthesis of PDPA-SS-KLA, PDPA-TK-CVR, and PDPA-TK-PEG_{5000}....................4

Preparation of GSH-PN, H_2O_2-PN, and MAPN...5

DLS and TEM analysis...6

Determination of CMCs..6

Stability of MAPN...6

CD spectra..7

Mitochondrial swelling...7

Cell viability assay..7

Cellular internalization and endosomal escape...8

In vitro PD-1/PD-L1 pathway blockade...9

The mtDNA leakage assay...9

The qPCR quantification of cytosolic mtDNA.. 9

Caspase-9 and Caspase-3 activity assay.. 10

Evaluation of cGAS-STING pathway activation...10

MAPN-mediated DCs maturation in vitro..11

In vivo distribution of MAPN..11

Tumor inhibition with MAPN..11

Statistical analysis..13

References..13

Supplemental figures and tables..15

Materials

Reagents and solvents were purchased from Sigma-Aldrich (Shanghai, China) and used as received without purification unless otherwise noted. SPDP and fluorescein isothiocyanate (FITC) were purchased from Alfa Aesar (Shanghai, China). 2,2'-(propane-2,2-diylbis(thio))diacetic acid and 2-maleimidoethylaminehydrochloride were purchased from Bide Pharmatech Ltd (Shanghai, China). Peptides were obtained fom Shanghai HongTide Biotechnology Co., Ltd.. Dialysis membranes were purchased from Tian Nan Science and Technology (Tianjin, China). B16F10 cells were purchased from American Type Culture Collection (ATCC). The Dulbecco's Modified Eagle Medium (DMEM) growth medium, RPMI-1640 growth medium, fetal bovine serum (FBS), 0.25% trypsin, and penicillin/streptomycin were purchased from Gibco (Thermo Fisher, USA). Cy5.5-NHS was purchased from Oukainasi Technology (Beijing, China). MeO-PEG_{5000}-NH_2 was purchased from Ponsure (Wuhan, China). Bicinchoninic acid (BCA) protein assay kit was obtained from Solarbio Science & Technology (Beijing, China). Multiplex PCR Kit and DNA Polymerase were purchased from Novoprotein Co., Ltd (Shanghai, China). Fluorescent TUNEL Staining Kit was purchased from Yeasen Biotech Co., Ltd. (Shanghai, China). dsDNA Marker was purchased from Santa Cruz Biotechnology (USA). All anti-bodies were purchased from Abcam (Shanghai, China), Biolegend (USA), Santa Cruz Biotechnology (USA), Servicebio (Wuhan, China), Elabscience Biotechnology. (Wuhan, China), Affinity Biosciences (Beijing, China), and BOSTER Biological Technology. (Wuhan, China). All ELISA kits was purchase from Jianglai biology (Shanghai, China).

Instruments

The particle sizes and zeta potentials of GSH-PN, H_2O_2-PN, and MAPN were studied by Zetasizer Nano-ZS (Malvern Instruments, USA) at 25 °C. Transmission Electron Microscopy (TEM) measurements were performed on a commercial Talos F200C electron microscope at an acceleration voltage of 120 kV. ^{1}H-nuclear magnetic resonance (^{1}H-NMR) spectra were obtained on a Varian UNITY-plus 600 M nuclear magnetic resonance spectrometer. UV-Visible spectra were performed on a ALPHA

FluorChem FC3 (Alpha, USA). Cell viability and BCA assay was reported on a Tecan Spark plate reader (USA). Flow cytometric analysis was performed on a CytoFLEX flow cytometry (Beckman, USA). CLSM images were captured with a Confocal Laser Scanning Microscopes-LSM 800 (Zeiss, Germany).

Synthesis and Characterization of PDPA-SS-KLA, PDPA-TK-CVR, and PDPA-TK-PEG_{5000}

To synthesize stimuli-responsive polymers, PDPA was first synthesized (detailed in **Figure S3a,** Supporting Information).[1] Briefly, 1.52 g (2.67 mmol) tpy-CTA, 5.69 g (26.7 mmol) DPA, and 43.9 mg (0.267 mmol) AIBN were dissolved in 60 mL dioxane. The solution was bubbled with argon gas for 45 min, then reaction system was sealed and stirred magnetically at 70 °C for 24 h. Subsequently, the reaction mixture was allowed to cool to room temperature followed by concentration, and dialysis (MWCO: 1000 Da) against methanol for 48 h. After removing the solvent by rotary evaporator, the obtained tpy-PDPA-CTA product was dried under vacuum, yielding 5.4 g (75 %). Then, 350 mg (0.1 mmol) tpy-PDPA-CTA, 38.9 mg (0.385 mmol) nhexylamine, and 3.86 mg (0.019 mmol) tributyl phosphine were dissolved in 4 mL THF. The solution was stirred at room temperature for 12 h at 25 °C, and dialysis (MWCO: 1000 Da) against methanol for 24 h. After removal of the solvent, the obtained PDPA-SH product was dried under vacuum. The PDPA-SH (100 mg, 0.028 mmol) was then reacted with N-(2-aminoethyl)maleimide (11.84 mg, 0.084 mmol) in PBS (pH 5.0, 10 mM), and further dialysis (MWCO: 1000 Da) against deionized water and lyophilized to obtaine PDPA-NH_2. The successful synthesis of PDPA-SH and PDPA-NH_2 were confirmed using ^{1}H NMR analysis as indicated in **Figure S4,** Supporting Information.

PDPA-SS-KLA was obtained through coupled reaction between PDPA-SPDP and SKLAKLAKKLAKLAK peptide. Briefly, PDPA-NH_2 (103.4 mg, 0.028 mmol) and SPDP (13.1 mg, 0.042 mmol) were added into PBS (pH 5.0, 10 mM), and the mixture was stirred at 4 ^{o}C for 4 h. The mixture was then dialyzed against PBS (pH 5.0, 10 mM) (MWCO: 1000 Da) and further reacted with SKLAKLAKKLAKLAK (84.8 mg, 0.042 mmol). The pH of mixture was then tuned to pH 7.4 and dialyzed against PBS (pH 7.4,

10 mM) (MWCO: 5000 Da) to remove unreacted peptides, and lyophilized to obtaine PDPA-SS-KLA. The successful synthesis of PDPA-SS-KLA was confirmed using gel permeation chromatography (GPC) and ^{1}H NMR as indicated in **Figure 2a** and **Figure S5**, Supporting Information.

PDPA-TK-CVR was obtained through amidation reaction between PDPA-TK and CVRARTR-$CONH_2$. Briefly, 2,2'-(propane-2,2-diylbis(thio))diacetic acid (28.3 mg, 0.084 mmol), EDC (14.36 mg, 0.126 mmol), and DMAP (15.23 g, 0.126 mmol) were dissolved in 5 mL DMF, and the mixture was stirred for 2 h at RT. PDPA-NH_2 (103.4 mg, 0.028 mmol) was added into the mixture and stirred for another 24 h. The mixture was precipitated into ice ether to obtain PDPA-TK. Then, EDC (10.69 mg, 0.056 mmol), NHS (6.44 mg, 0.056 mmol), PDPA-TK (106.1 mg, 0.028 mmol) were dissolved in PBS (pH 5.0, 10 mM), and the mixture was stirred for 2 h at RT. CVRARTR-$CONH_2$ (30.56 mg, 0.042 mmol) was added into the mixture and stirred for another 24 h. The pH of mixture was tuned to pH 7.4 and dialyzed against PBS (pH 7.4, 10 mM) (MWCO: 5000 Da) to remove the unreacted peptides, and lyophilized to obtaine PDPA-TK-CVR. PDPA-TK-PEG_{5000} was synthesized using a similar method by replacing CVRARTR-$CONH_2$ with MeO-PEG_{5000}-NH_2. The successful synthesis of PDPA-TK-CVR and PDPA-TK-PEG_{5000} were confirmed using GPC and ^{1}H NMR as indicated in **Figure 2a** and **Figure S6**, Supporting Information.

For the better demonstration, PDPA-KLA, PDPA-CVR, and PDPA-PEG_{5000} were synthesized as nonresponsive polymers using similar methods as indicated in **Figure S8**, Supporting Information. The successful synthesis of PDPA-KLA, PDPA-CVR, and PDPA-PEG_{5000} were confirmed using GPC and ^{1}H-NMR as indicated in **Figure S9** and **Figure S10a**, Supporting Information.

Preparation of GSH-PN, H_2O_2-PN, and MAPN

To prepare MAPN, PDPA-SS-KLA (20 μmol), PDPA-TK-CVR (20 μmol), and PDPA-TK-PEG_{5000} (20 μmol) were first dissolved in 0.5 mL DMSO, and then slowly dropped into PBS (pH 7.4, 10 mM) at 25 °C. DMSO was then removed by dialysis (MWCO: 5000 Da). GSH-PN was prepared with PDPA-SS-KLA, PDPA-CVR, and

PDPA-PEG_{5000} using a similar method. H_2O_2-PN was prepared with PDPA-KLA, PDPA-TK-CVR, and PDPA-TK-PEG_{5000} using a similar method.

DLS and TEM Analysis

The average particle sizes and zeta potentials of GSH-PN, H_2O_2-PN, and MAPN were determined using DLS measurements. The DLS measurements were performed on a Zetasizer Nano-ZS (Malvern Instruments, USA) at 37 °C. The morphology of GSH-PN, H_2O_2-PN, and MAPN were observed using transmission electron microscopy (TEM, FEI Talos F200C electron microscope). For the preparation of TEM samples, GSH-PN, H_2O_2-PN, and MAPN were prepared as the solutions with pH 7.4 and the concentration of KLA at 4 μM. TEM samples were prepared by drop-coating of 2 μL GSH-PN, H_2O_2-PN, and MAPN onto carbon-coated copper grids. Droplets of samples were contacted with the grids for 15 min, then excess amount of samples was removed. The grids were then rinsed and stained with 1% sodium uranyl acetate (5~10 μL) for 120 s.

Determination of Critical Micelle Concentration (CMC)

PDPA-SS-KLA, PDPA-TK-CVR, and PDPA-TK-PEG_{5000} can self-assemble into micelle at pH 7.4, with PDPA as the hydrophobic inner core, PEG and peptides as the hydrophilic outer corona. The CMC values of PDPA-SS-KLA, PDPA-TK-CVR, and PDPA-TK-PEG_{5000} were determined by fluorescence spectroscopy using pyrene as a fluorescence probe at pH 7.4.[2] An aliquot of 1 mL acetone solution of pyrene was transferred into 20 mL vials and the acetone evaporated to dryness. Then, a series of PDPA-SS-KLA, PDPA-TK-CVR, or PDPA-TK-PEG_{5000} micellar solutions ranging from 1×10^{-5} to 1 mg mL^{-1} were added into the vials to give a final pyrene concentration of 6×10^{-7} M. The solution was incubated at room temperature overnight under shaking. The measured emission wavelength was set at 395 nm, and the band width of emission was set to 3 nm. The ratios of the excitation spectra's fluorescent intensities at 337 nm and 335 nm (I_{337}/I_{335}) were calculated and plotted against the logarithm of polymer mass concentration.

Stability of MAPN

The stability of MAPN was evaluated using previous method.[3] Briefly, we dissolved the MAPN with a concentration of KLA at 4 μM in PBS (pH 7.4, 10 mM) and mouse serum at 37 °C, and the particle sizes of MAPN were characterized at various incubation time using DLS measurement.

Circular Dichroism (CD) Spectra

MAPN and H_2O_2-PN were pretreated with GSH (10 mM) to simulate the GSH-riched intracellular environment, and incubated in 10% (v/v) solution of trifluoroethanol/PBS to imitate the membrane environment.[4] CD experiments were performed with a Bio-Logic MOS-450 (Claix, France) at room temperature. The experimental condition was set as follows: speed of 50 nm/min, time response of 2s, resolution of 0.5 nm, bandwidth of 4.0 nm, and cell path length of 1.0 mm. All spectra were converted to a uniform scale after subtraction of the background. The recorded curves were smoothed with standard parameters.

Mitochondrial Swelling

MAPN and H_2O_2-PN were pretreated with GSH (10 mM) to simulate the GSH-riched intracellular environment. The mitochondria were isolated using a cell mitochondria isolation kit (Beyotime, China). 5×10^6 B16F10 cells were collected and suspended with 2.5 mL mitochondrial separation reagent. The cell suspension was placed in ice bath for 15 min and then transferred to a glass homogenizer for 20 times' homogenate. The cell homogenate was centrifuged at 600 g for 10 min. The supernatant was carefully transferred to another centrifuge tube and centrifuged at 11000 g for 10 min. The precipitate is the isolated cell mitochondria. The isolated mitochondria were suspended in storage buffer at a concentration of 0.5 mg mL^{-1}. 100 μL of different samples were added to above mitochondrial suspension and absorbance at 540 nm was continuously scanned within 15 min.[5]

Cell Culture

B16F10 and BMDC cells were cultured in RAPI-1640 supplemented with 10% (v/v) heat-inactivated FBS, 100 units mL^{-1} penicillin, and 100 μg mL^{-1} streptomycin, and maintained in 5% CO_2 humidified environment at 37 °C.

Cell Viability Assay

The cell viability assay was evaluated using Cell Counting Kit (CCK, ZOMANBIO Biotech, Beijing, China) as previously described.[3] Briefly, B16F10 cells were seeded in a 96-well plate at a density of 5×10^3 cells per well and incubated overnight in 0.5 mL RAPI-1640 with 10% FBS (v/v) for overnight. The cells was exposed to free KLA, H_2O_2-PN, and MAPN with or without H_2O_2 (100 μM) at various KLA concentrations (from 1 to 32 μM). Then, CCK reagent was mixed with RPMI-1640 at a volume ratio of 1/9 (freshly prepared) to achieve the CCK working solution. After 24 h incubation, the cells were rinsed using PBS (pH 7.4, 10 mM), followed by the addition of 100 μL CCK working solution for another 2 h incubation. Quantification of cell viability was determined by measuring the absorbance (λ = 450 nm) with Synergy H1 microplate reader (BIO-TEK, USA). The cell viability was calculated by referring to that of the cells without any treatment.

Cellular Internalization and Endosomal Escape

Cellular internalization of MAPN was studied through CLSM and flow cytometric assays (Beckman, USA). Briefly, B16F10 cells were seeded at a density of 1×10^4 cells per well in 35 mm confocal dish (Φ =15 mm) and incubated overnight for the cell attachment. The cells were incubated with free KLA, GSH-PN, and MAPN with a concentration of KLA at 4 μM in complete culture medium with or without H_2O_2 (100 μM) for 4 h. The cells were then washed twice with ice-cold PBS and fixed with fresh 4% paraformaldehyde at room temperature, and then counterstained with DAPI. All the cells were observed using a CLSM (Zeiss, Germany). For flow cytometric analysis, B16F10 cells were seeded into 6-well plates at a density of 1×10^5 cells per well. After the overnight growth, the cells were exposed to frre KLA, MAPN, and GSH-PN with a concentration of KLA at 4 μM, and incubated in complete culture medium at different conditions (with or without H_2O_2, 100 μM) for 2 h. After the trypsin digestion and centrifugation, the cells were collected, washed with cold PBS and fixed with fresh 4% paraformaldehyde for flow cytometric assay. All of these experiments were performed in triplicate.

The endosomal escape ability of MAPN was study by analyzing the colocolization of endosomes and KLA after internalizing into cells. Briefly, B16F10 cells were seeded at a density of 1×10^4 cells per well in 35 mm confocal dish (Φ =15 mm) and incubated overnight for cell attachment. KLA was labeled with FITC. The cells were incubated with MAPN with a concentration of KLA at 4 μM in complete culture medium with H_2O_2 (100 μM) for 4 h. After 1 h, 3 h, and 6 h incubation, endosomes were stained with LysoTracker probe (red) according to manufacture's instruction. The cells were then washed twice with ice-cold PBS and fixed with fresh 4% paraformaldehyde for 15 min at room temperature, and counterstained with DAPI for the easy observation of cell nucleus. All the cells were observed using a CLSM (Zeiss, Germany).

In Vitro PD-1/PD-L1 Pathway Blockade

MAPN-mediated PD-1/PD-L1 pathway blockade was evaluated with anti-PD-L1 (sc-518027, Santa Cruz, USA) staining. Briefly, B16F10 cells were seeded at a density of 1×10^4 cells per well in 35 mm confocal dish (Φ =15 mm) and incubated overnight for cell attachment. The cells were incubated with MAPN with a concentration of KLA at 4 μM in complete culture medium with or without H_2O_2 (100 μM) for 4 h. The cells were then washed twice with ice-cold PBS and fixed with fresh 4% paraformaldehyde at room temperature, and then stained with Alexa Fluor 594 (red) labeled anti-PD-L1 antibodies and DAPI. All the cells were observed using a CLSM (Zeiss, Germany).

The mtDNA Leakage Assay

B16F10 cells were seeded at a density of 1×10^4 cells per well in 35 mm confocal dish (Φ =15 mm) and incubated overnight for cell attachment. The cells were incubated with MAPN with a concentration of KLA at 4 μM in complete culture medium with or without H_2O_2 (100 μM) for 24 h. After washing with PBS, the cells were permeabilized, and incubated with Anti-TOMM20 antibodies (GB111481, Servicebio, China) and dsDNA Marker (sc-58749, Santa Cruz, USA) at 4 °C overnight, and then incubated with secondary antibodies for 2 h and counterstained with DAPI. All the cells were observed using a CLSM (Zeiss, Germany).

The qPCR Quantification of Cytosolic mtDNA

B16F10 cells were seeded in a 12-well plate at a density of 5×10^4 cells per well and incubated overnight in 1 mL RAPI-1640 with 10% FBS (v/v) for overnight. The cells was exposed to free KLA, H_2O_2-PN, and MAPN with a concentration of KLA at 4 μM in complete culture medium with or without H_2O_2 (100 μM) for 24 h. After washing with PBS, the cells were collected by centrifugation. Cytosolic mtDNA was removed using a Cell Mitochondria Isolation Kit (C3601, Beyotime, China), and the DNA was then extracted with a TIANamp Genomic DNA kit (DP304, Tiangen Biotech, China) following the manufacturer's protocol. The mtDNA D-Loop primers were synthesized by GenePharma Biotech (detailed sequences in **Table S3**, Supporting Information), and then quantified with Multiplex PCR Kit (PM101, Novoprotein , Shanghai, China) using Quantitative Real-Time PCR Detection System (Bio-Rad, CFX96, USA).

Caspase-9 and Caspase-3 Activity Assay

The B16F10 cells were seeded into a 6-well plate at the density of 2×10^5 cells per well. After the overnight growth, the cells were exposed to frre KLA, MAPN, and GSH-PN with a concentration of KLA at 4 μM, and incubated in complete culture medium at different conditions (with or without H_2O_2, 100 μM) for 24 h. After treatments, the cells were rinsed with PBS for three times and then treated with lysis buffer solution in ice bath for 1 h. Cell lysates were centrifuged at 12000 rpm for 5 min at 4 ^oC, and the supernatant was collected. The supernatant was incubated with caspase-9 or caspase-3 substrate at 37 ^oC in dark for 12 h. The activities of caspase-9 and caspase-3 were determined by measuring the absorbance (λ = 450 nm) with Synergy H1 microplate reader (BIO-TEK, USA).

Evaluation of cGAS-STING Pathway Activation

Western bolt was employed to evaluate the MAPN-mediated activation of cGAS-STING pathway. Briefly, B16F10 cells were first seeded into 24-well plates at 2×10^4 cells per well and incubated overnight in 0.5 mL RAPI-1640 with 10% FBS (v/v) for cell attachment. After the overnight growth, the cells were exposed to frre KLA, GSH-PN, and MAPN with a concentration of KLA at 4 μM, and incubated in complete culture medium with or without H_2O_2 (100 μM). After 24 h incubation, the cells were

washed with ice-cold PBS for three times and solubilized in 1% Nonidet P-40 lysis buffer. Homogenates were clarified by centrifugation at 15000 g for 30 min at 4 °C, and protein concentrations were determined with a BCA assay kit (Solarbio, China). Total protein lysates were separated by SDS-PAGE on 10% SDS acrylamide gels, then transferred to PVDF membranes (Millipore, USA). The membranes were incubated with primary antibodies against p-STING (AF7416, Affinity Biosciences), STING (ab179775, Abcam), p-TBK1 (AF8190, Affinity Biosciences), TBK1 (sc-52957, Santa Cruz), p-IRF3 (4947S, Cell Signaling Technology), and IRF3 (sc-33641, Santa Cruz) (1:1000 dilution; Abcam, USA) overnight at 4 °C, followed by incubating with an HRP-conjugated secondary antibodies (1:2000 dilution; Boster Biological Technology Co. Ltd, China) for 1 h. GAPDH (BM1623, Boster Biological Technology Co. Ltd, China) was set as a loading control.

MAPN-Mediated DCs Maturation *In Vitro*

To investigate the MAPN-mediated DCs maturation, BMDCs were first extracted from the bone marrow of 8-week-old C57BL/6 mice. B16F10 cells were pretreated with PBS, free KLA, H_2O_2-PN, and MAPN as described above. Afterwards, 1×10^6 immature BMDCs were co-cultured with 1×10^5 pretreated B16F10 cells for 24 h using a transwell chamber (The B16F10 cells were cultured in the upper chamber, and BMDCs were seeded in the lower chamber). After staining with anti-CD45, anti-CD11c, anti-CD80, and anti-CD86 antibodies according to the manufacturer' protocols, the DCs maturation was examined by flow cytometric measurement.

In Vivo Distribution of MAPN

To investigate the tumor accumulating ability of MAPN, tumor-bearing mice were established by subcutaneous injection of B16F10 cells (1×10^6 for each mouse) in the mammary fat pad. The mice were randomly divided into three groups (n = 3). When the tumor volume was about 500 mm^3, the mice were intravenously injected with free KLA, GSH-PN, and MAPN containing Cy5.5 labeled KLA (100 µM; 150 µL). All experimental protocols were conducted within Anhui Medical University guidelines for animal research and were approved by Institutional Animal Care and Use Committee

(LLSC20230877). At 24 h post-injection, the mice were imaged by IVIS Lumina imaged system (IVIS, Spectral Instrumen, USA). The mice were then sacrificed, and major organs and tumors were collected for *ex vivo* imaging. The fluorescence images were analyzed using Version 4.0.0 (Spectral Instrumen).

Tumor Inhibition with MAPN

To investigate the anti-tumor effects of MAPN treatment, B16F10 tumor-bearing mice was established as described above. When primary tumors reached a size of about 30-60 mm^3, the mice were randomly divided into five groups (n = 6), and intravenously injected with PBS, free KLA, GSH-PN, H_2O_2-PN, and MAPN (KLA: 100 μM, 150 μL) every four days for four times. The body weights and tumor volumes were measured every four days. Tumor volumes were calculated with the formula: tumor volume = 0.5 × length × $width^2$. The mice were sacrificed when the volume of tumor exceeded 2 cm^3. About one-half of the mice were sacrificed on day 15 after tumor implantation, the blood and major organ of mice were collected and analyzed. Survival was evaluated from the first day of tumor implantation until day 42. To examine the immune responses against primary tumor, $CD45^+$ $CD3^+$ $CD8^+$ T cells, $CD45^+$ $CD11c^+$ $CD86^+$ $CD80^+$ DCs, $ki67^+$ $CD8^+$ T cells, and IFN-γ^+ $CD8^+$ T cells in tumors and LNs were analyzed using flow cytometry. All anti-bodies for flow cytometric analysis were purchased from Biolegend (USA). IFN-β, IFN-γ, TNF-α, IL-12, and IL-6 in tumors were also examined with ELISA kits (Jianglai biology, Shanghai). In apoptotic cells, the double or single strand breaks of chromosome DNA could produce 3’-OH terminal. With catalysis of deoxyribonucleotide terminal transferase, dUTP with fluorescein molecule was labeled to the 3’-terminal of DNA, and then observed by fluorescence microscope to detect cell apoptosis. For deoxynucleotidyl transferase dUTP nick end labeling (TUNEL) studying, the fixed tumor sections were stained using a TUNEL Apoptosis Assay Kit (40307ES20, Yeasen Biotech, China) according to the manufacturer’s protocol.

To further demonstrate the effective activation of T cell anti-tumor immunity after MAPN treatment, we examined the anti-tumor effects of MAPN in a $CD8^+$ T cell-depleted B16F10 tumor-bearing model. Briefly, when tumors reached a size of about

30-60 mm^3, tumors were first intratumorally injected with αCD8 at a dose of 2.5 mg kg^{-1} (αCD8; BioLegend, USA). Next day, the mice was intravenously injected with PBS and MAPN as described above. The tumor volumes was continuously monitored during four rounds treatments.

To investigate the potential of MAPN in inducing long-term immune memory effects, we constructed a rechallenged tumor model as described in previous study.[3] Briefly, 1×10^6 B16F10 cells were transplanted into the right frank of C57BL/6 mice. When tumors reached a size of about 30-60 mm^3, mice were randomly divided into five groups (n = 6) and intravenously injected with PBS, free KLA, GSH-PN, H_2O_2-PN, and MAPN (KLA: 100 μM, 150 μL). After three times treatments, primary tumors in the right flank were completely removed surgically, and then rechallenged with B16F10 cells (1×10^6 cells) in the left flank. The mice were then sacrificed and the metastatic tumor nodules in the surface of lung were counted and analyzed using H&E staining on day 27 post-rechallenge. To determine the memory T cells, spleens were harvested and stained with anti-CD3, anti-CD8, anti-CD44, and anti-CD62L according to manufacturer' protocols. The effector memory T cells (T_{EM}, $CD3^+$ $CD8^+$ $CD44^+$ $CD62L^-$ cells), central memory T cells (T_{CM}, gated on $CD3^+$ $CD8^+$ $CD44^+$ $CD62L^+$ cells) were analyzed using flow cytometry.

Statistical Analysis

All results are expressed as the standard deviation (SD) or standard error of mean (SEM) as indicated. Statistical comparisons were achieved *via* the Student's t-test or one-way ANOVA with Tukey's post-hoc test. All statistical analyses were carried out using GraphPad Prism 8.0. The significant levels are shown as $^*p < 0.05$, $^{**}p < 0.01$, and $^{***}p < 0.001$ as indicated.

References

[1] L. Yan, Y. Liu, Z. Wang, H. Liu, *Macromol. Chem. Phys.* **2023**, *224*, 2200321.

[2] W. Wu, W. Wang, S. Li, J. Wang, Q. Zhang, X. Li, X. Luo, J. Li, *J. Polym. Res.* **2014**, *21*, 494.

[3] Y. Xing, J. Yang, Y. Wang, C. Wang, Z. Pan, F.-L. Liu, Y. Liu, Q. Liu, *Acs Nano* **2023**, *17*, 5713.

[4] a) J. Lee, E.-T. Oh, H. Lee, J. Kim, H. G. Kim, H. J. Park, C. Kim, *Bioconjugate Chem.* **2020**, *31*, 43; b) H. Sun, W. Choi, N. Zang, C. Battistella, M. P. Thompson, W. Cao, X. Zhou, C. Forman, N. C. Gianneschi, *Angew. Chem. Int. Edit.* **2019**, *58*, 17359.

[5] Z. Kang, C. Wang, Z. Zhang, Q. Liu, Y. Zheng, Y. Zhao, Z. Pan, Q. Li, L. Shi, Y. Liu, *Adv. Mater.* **2022**, *34*, 2201945.

Supplemental Figures and Tables

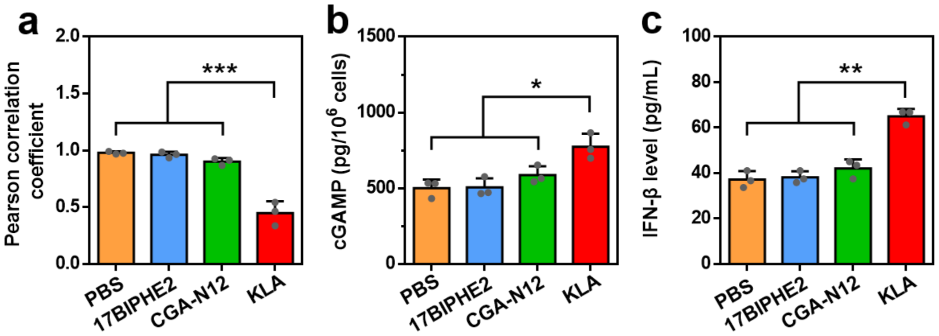

Figure S1. a) Pearson correlation coefficient in mmunofluorescence images of B16F10 cells treated with different AMPs (n = 3). b, c) Secretion levels of cGAMP (b) and IFN-β (c) in the B16F10 cells after different treatments (n = 3). Data are mean ± standard deviation (SD). Statistical significances were calculated *via* the Student's t-test (*$^*p < 0.05$, $^{**}p < 0.01$, $^{***}p < 0.001$*).

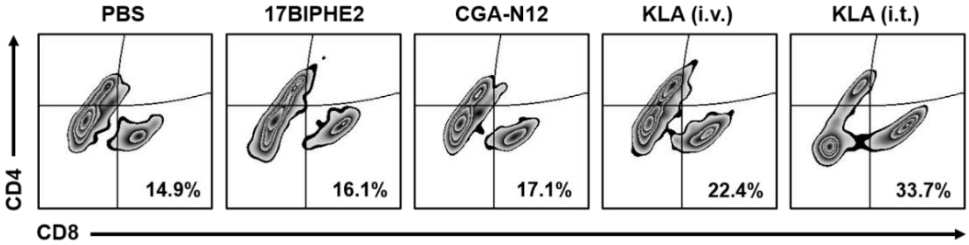

Figure S2. Flow cytometric analysis of $CD8^+$ CTLs infiltration (gated on $CD45^+$ $CD3^+$ $CD8^+$ cells) in tumor tissues after different treatments.

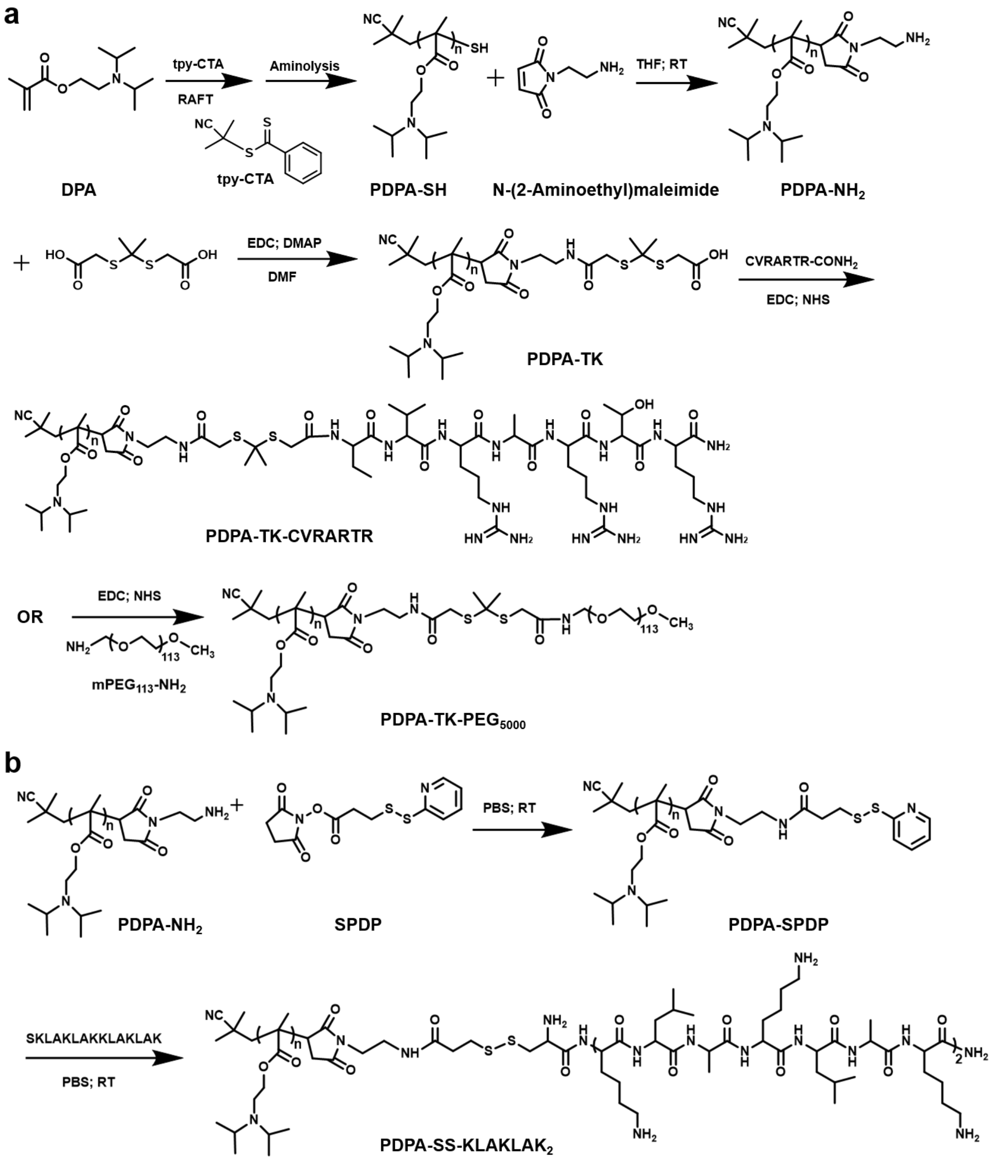

Figure S3. Schematic illustration of the synthesis of PDPA-TK-CVR and PDPA-TK-PEG$_{5000}$ (a), and PDPA-SS-KLA (b).

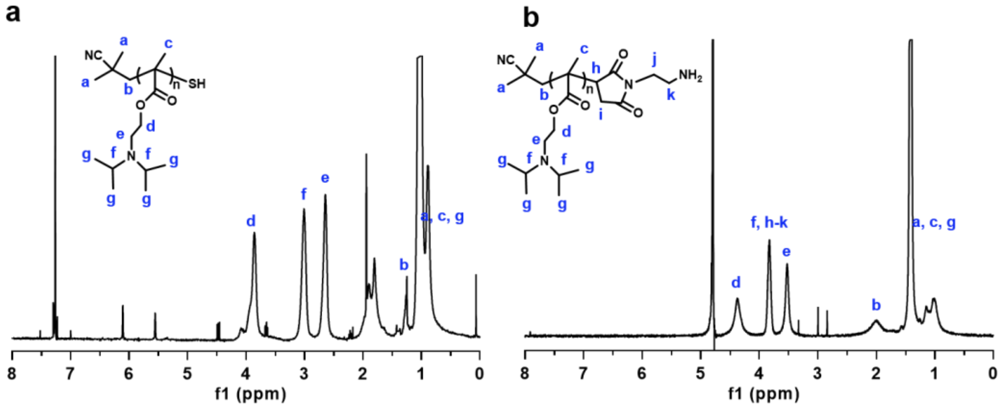

Figure S4. ^{1}H NMR spectra of PDPA-SH (a), and PDPA-NH_2 (b) (D_2O, 600 MHz).

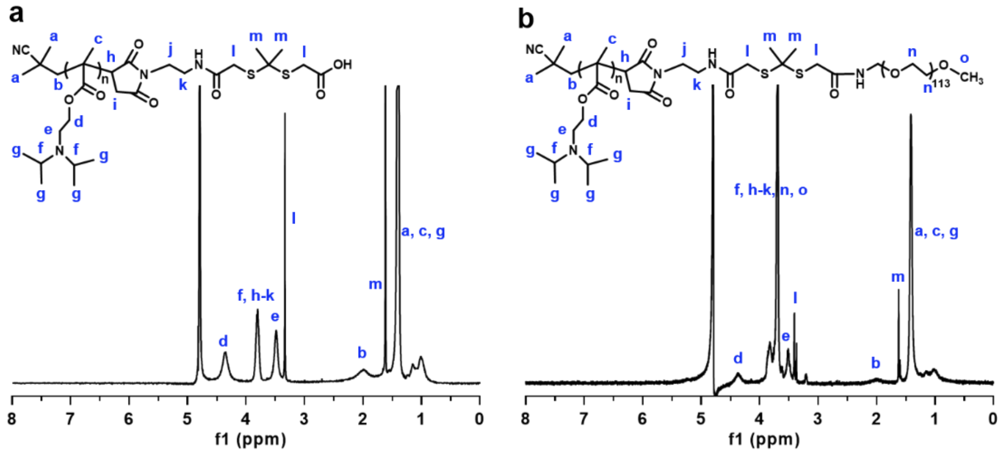

Figure S5. ^{1}H NMR spectra of PDPA-TK (a), and PDPA-TK-PEG_{5000} (b) (D_2O, 600 MHz).

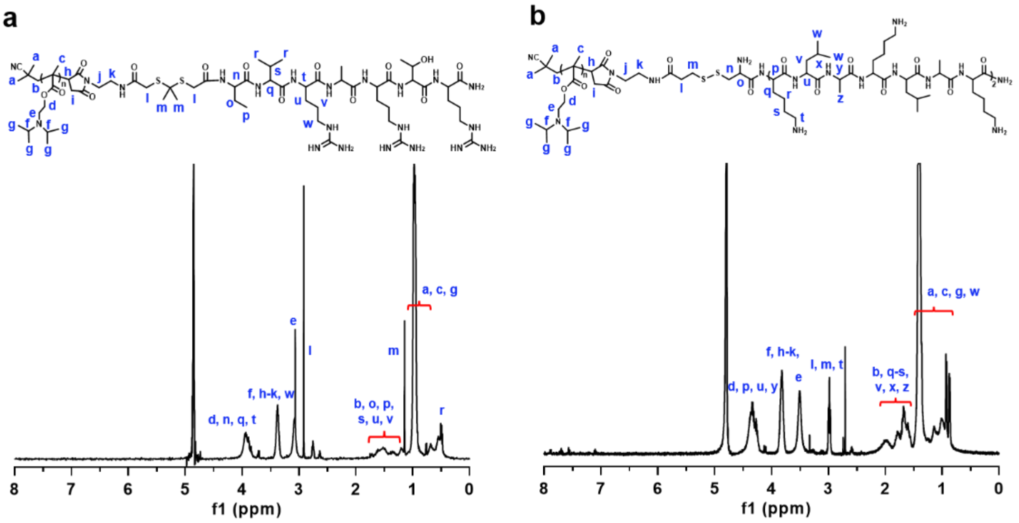

Figure S6. ^{1}H NMR spectra of PDPA-TK-CVR (a), and PDPA-SS-KLA (b) (D_2O, 600 MHz).

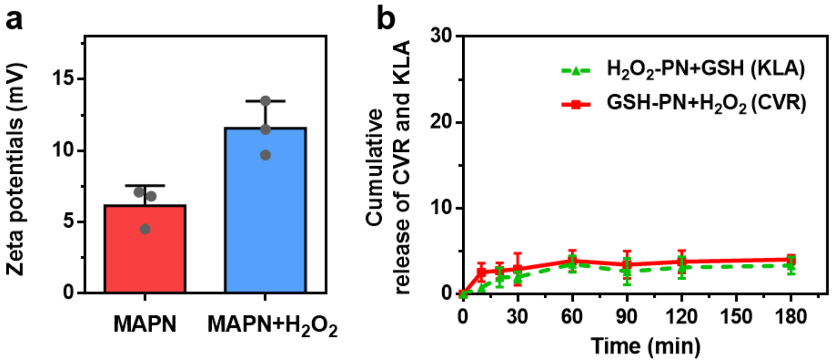

Figure S7. a) Zeta potentials of MAPN and MAPN+H_2O_2 (n = 3). b) Cumulative release of CVR and KLA from GSH-PN+H_2O_2 and H_2O_2-PN+GSH, respectively (n = 3). Data are mean ± standard deviation (SD).

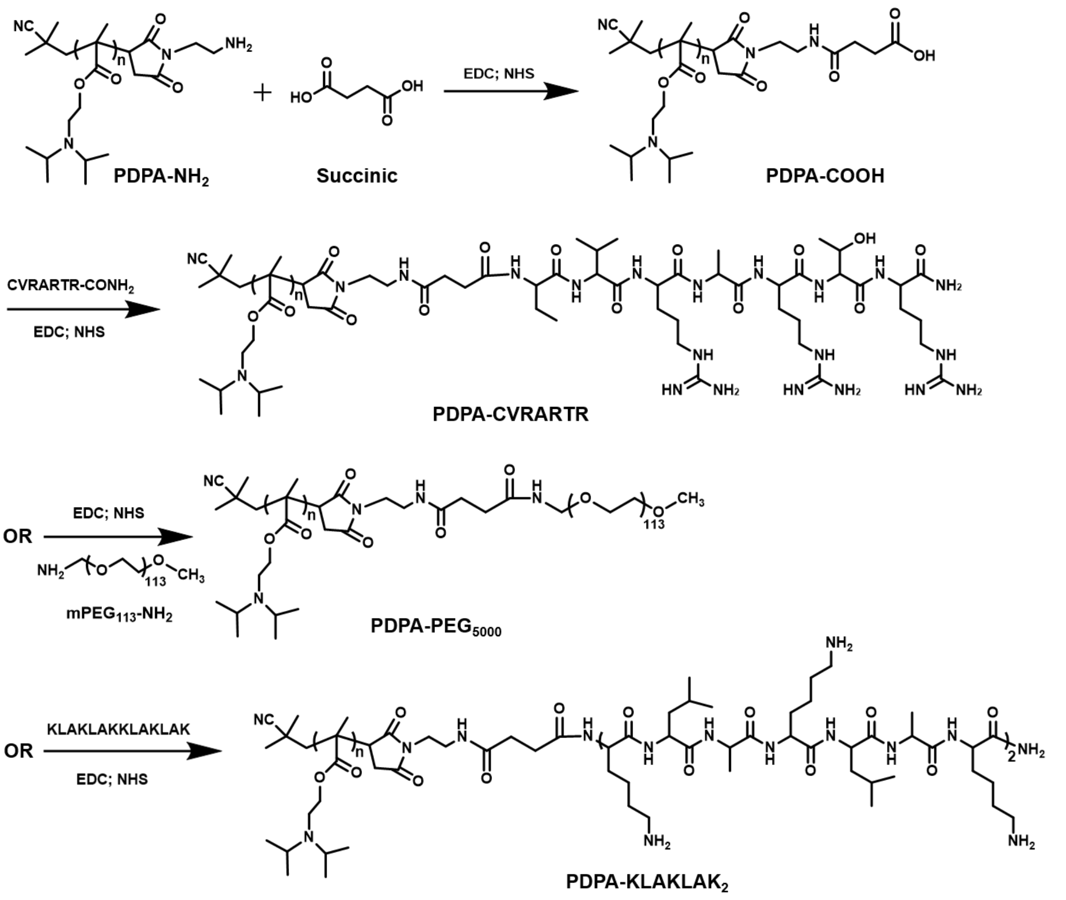

Figure S8. Schematic illustration of the synthesis of PDPA-CVR, PDPA-PEG_{5000}, and PDPA-KLA.

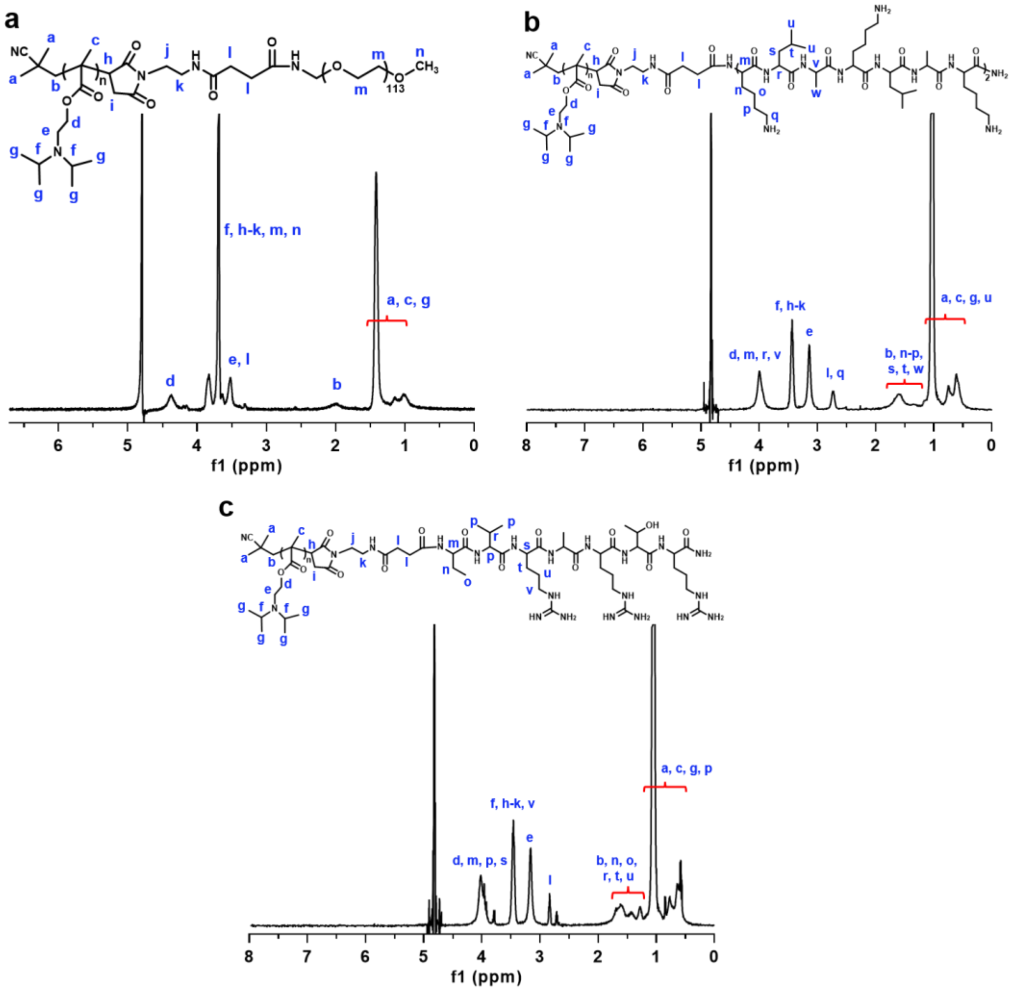

Figure S9. ^{1}H NMR spectra of PDPA-PEG_{5000} (a), PDPA-KLA (b), and PDPA-CVR (c) (D_2O, 600 MHz).

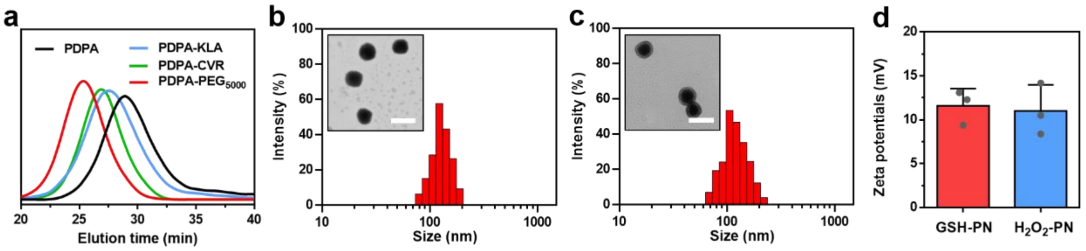

Figure S10. a) GPC analysis of free PDPA, PDPA-PEG_{5000}, PDPA-CVR, and PDPA-KLA. b, c) TEM and DLS measurements of GSH-PN (b) and H_2O_2-PN (c) at pH 7.4. Sclar bars are 200 nm. d) Zeta potentials of GSH-PN and H_2O_2-PN (n = 3). Data are mean ± standard deviation (SD).

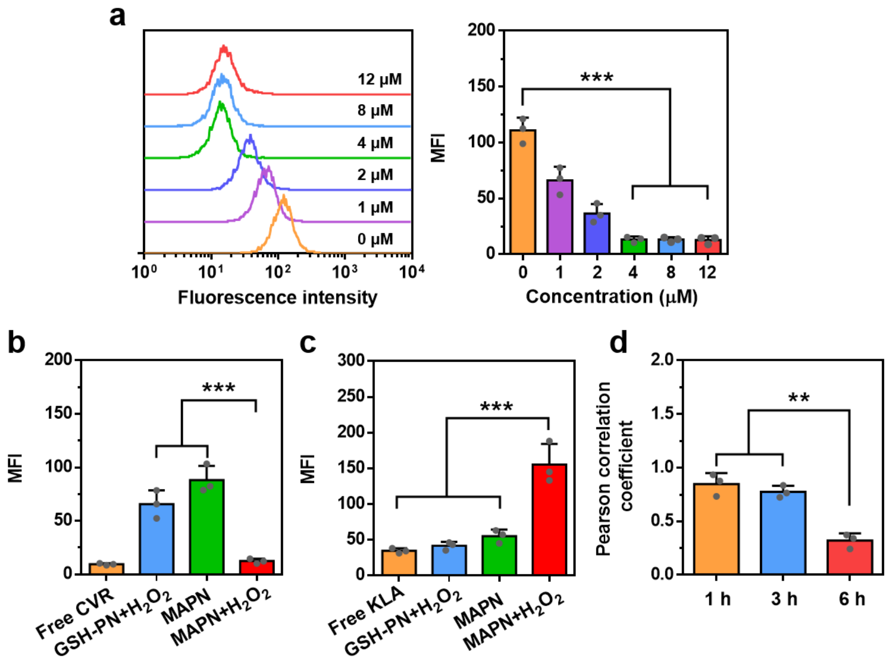

Figure S11. a) Flow cytometric analysis of anti-PD-L1-incubated B16F10 cells after pre-treated with free CVR at various concentrations for 2 h at 37 °C. Anti-PD-L1 was labeled with Alexa Fluor 594 (red). b) Mean fluorescence intensity (MFI) of anti-PD-L1-incubated B16F10 cells after pre-treated with free CVR, GSH-PN, or MAPN under different conditions for 2 h at 37 °C. Anti-PD-L1 was labeled with Alexa Fluor 594 (red) (n = 3). c) B16F10 cells after incubated with free KLA, GSH-PN, or MAPN under different conditions for 4 h at 37 °C. KLA was labeled with FITC (green) (n = 3). d) Pearson correlation coefficient of B16F10 cells treated with MAPN+H_2O_2 for 1 h, 3 h, and 6 h. Endosomes were stained with LysoTracker probe (red). KLA was labeled with FITC (green) (n = 3). Data are mean ± standard deviation (SD). Statistical significances were calculated *via* the Student's t-test (*$**p < 0.01$*, *$***p < 0.001$*).

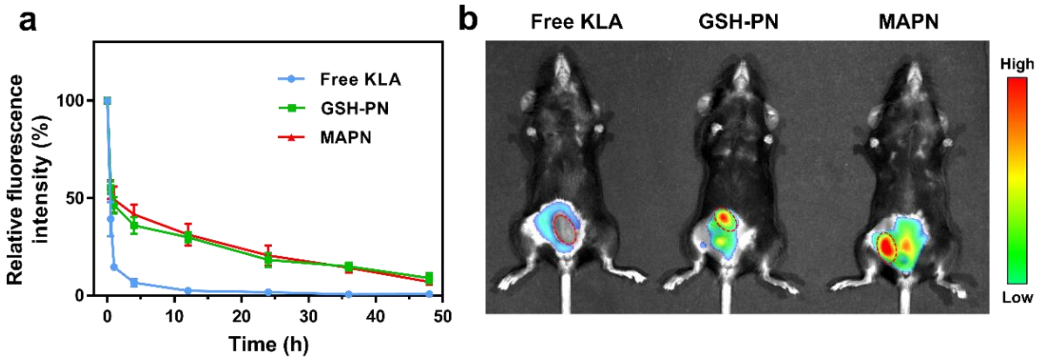

Figure S12. a) *In vivo* pharmacokinetics of free KLA, GSH-PN, and MAPN within 48 h. KLA was labeled with Cy5.5 (n = 3). b) IVIS images of B16F10-tumor bearing mice after intravenous injection of free KLA, GSH-PN, and MAPN at 24 h post-injection. KLA was labeled with Cy5.5.

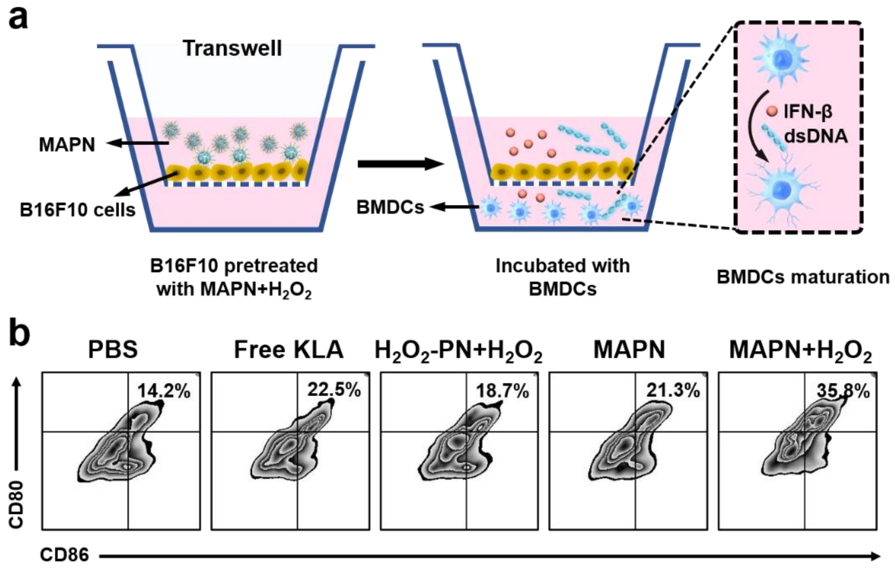

Figure S13. a) Schematic illustration of transwell experiment. The B16F10 cells were cultured in the upper chamber, and BMDCs were seeded in the lower chamber. b) Representative plots of matured BMDCs (gated on $CD11c^+$ $CD80^+$ $CD86^+$ cells) in each group analyzed by flow cytometry.

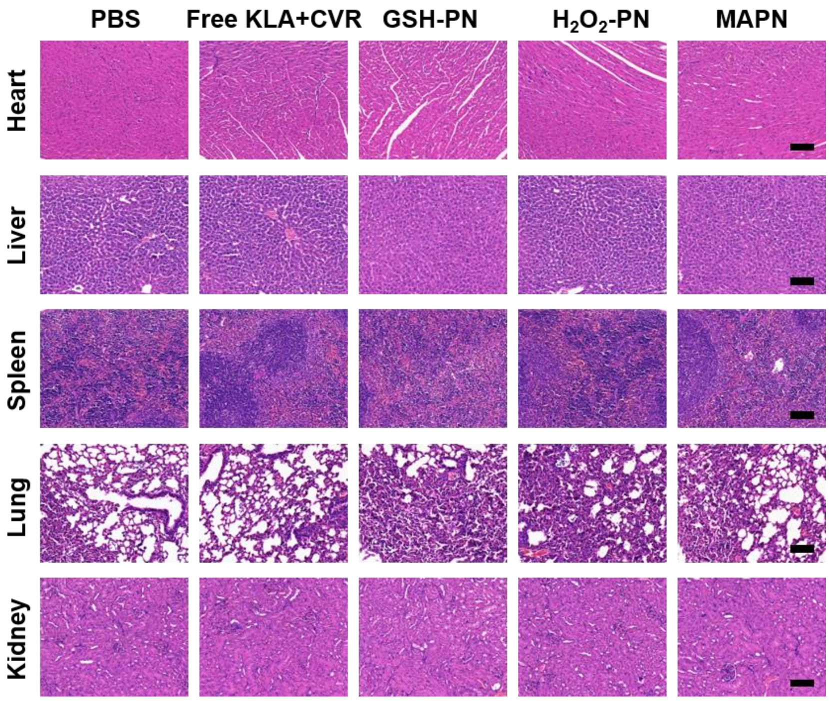

Figure S14. H&E analysis of the major organs (heart, liver, spleen, lung, and kidney) from the mice in each treatment group. The scale bars are 100 μm.

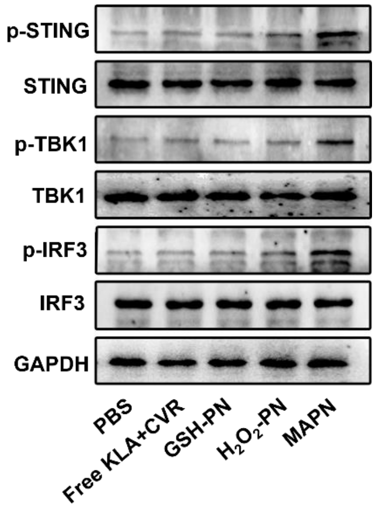

Figure S15. Western bolt images of p-STING, STING, p-TBK1, TBK1, p-IRF3, and IRF3 protein levels in tumor tissues after different treatments.

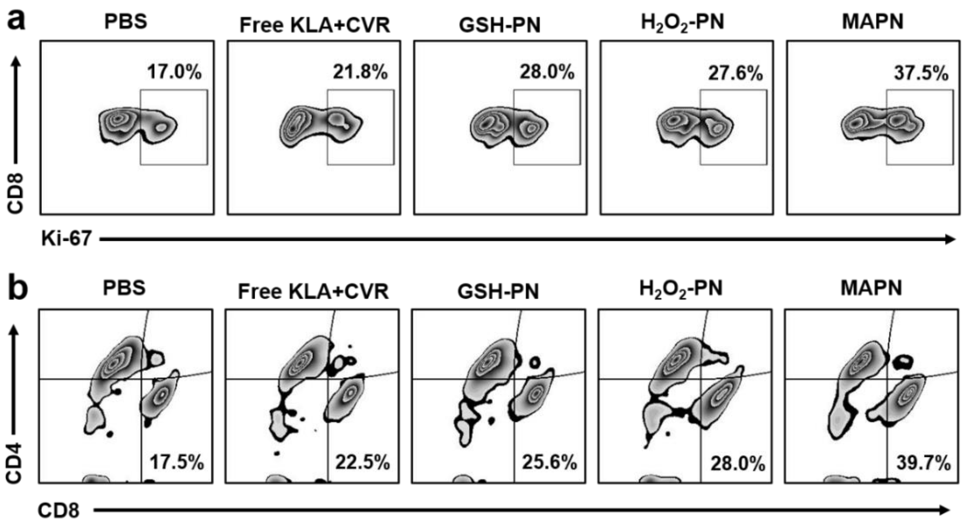

Figure S16. a) Representative flow cytometric plots of IFN-γ$^+$ CD8$^+$ CTLs in tumor tissues after different treatments. b) Representative flow cytometric plots of CD8$^+$ CTLs infiltration in spleens after different treatments.

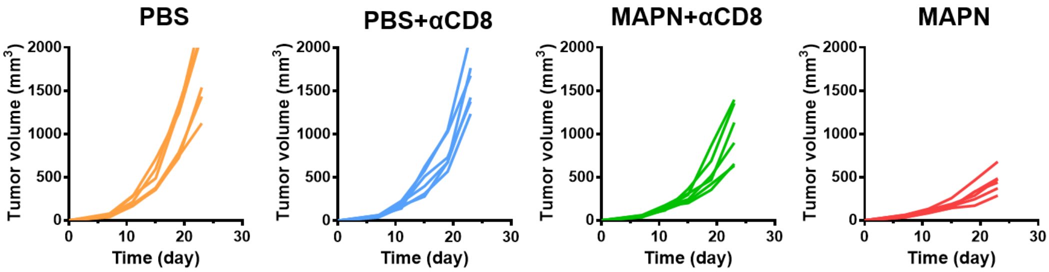

Figure S17. Individual tumor growth curves of B16F10 tumor-bearing mice after different treatments.

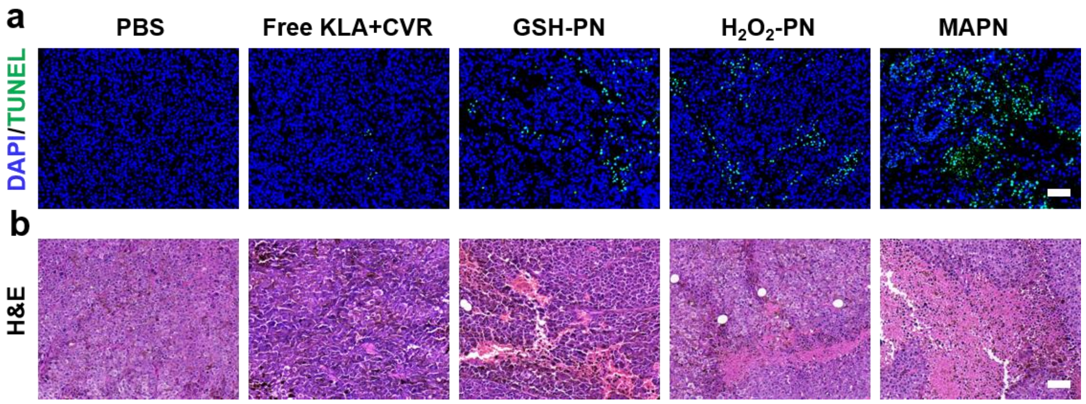

Figure S18. TUNEL (a) and H&E (b) staining of tumor tissues from the mice after different treatments. Scale bars are 100 μm.

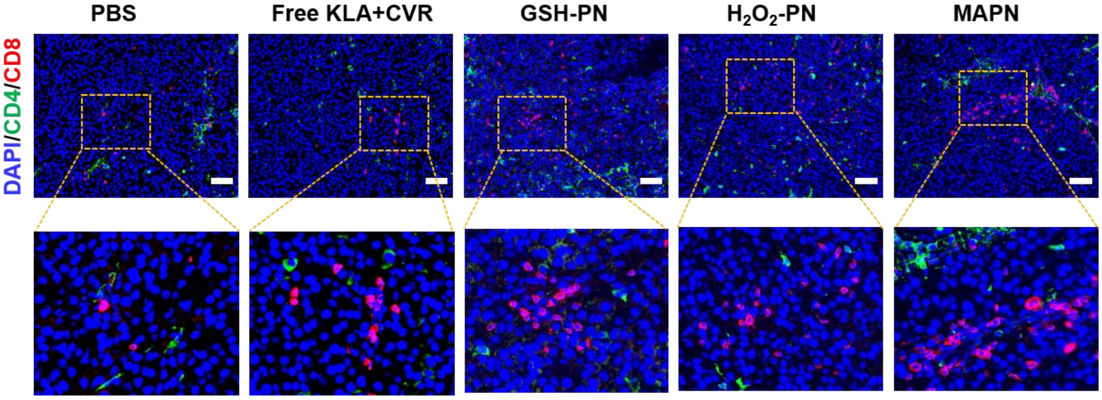

Figure S19. Immunofluorescence analysis of $CD4^+$ (green) and $CD8^+$ (red) CTLs infiltration in tumor tissues from the mice after different treatments. Nuclei was stained with DAPI (blue). Scale bars were 100 μm.

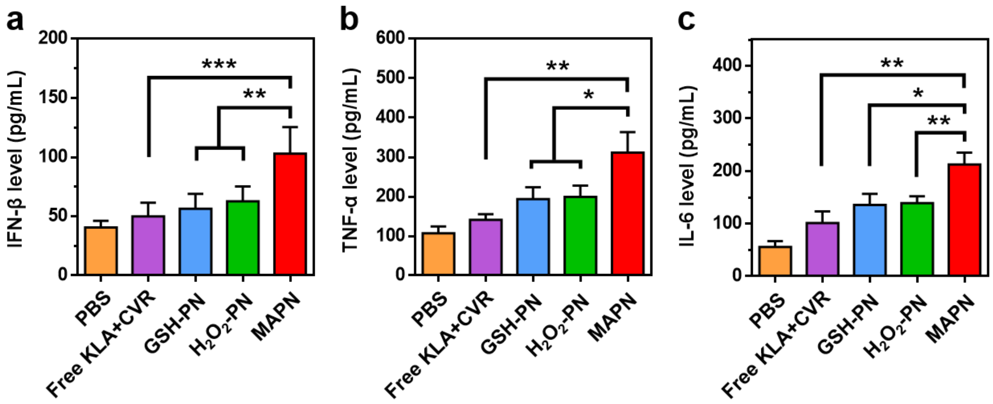

Figure S20. Relative levels of IFN-β (a), TNF-α (b), and IL-6 (c) in the rechallenged tumors from the mice after different treatments (n = 3). Data are presented as mean ± standard deviation (SD). Statistical significance were calculated *via* the Student's t test ($^*p < 0.05$, $^{**}p < 0.01$, $^{***}p < 0.001$).

Table S1. Sequences of peptides.

Peptide name	Sequence (N-C)
KLA	KLAKLAKKLAKLAK
CVR	CVRARTR
17BIPHE2	GBKRLVQRLKDBLRNLV
CGA-N12	ALQGAKERAHQQ

Table S2. The size and zeta potential of H_2O_2-PN, GSH-PN, and MAPN.

	**Zeta potential**	**Zeta potential in the presence of H_2O_2**	**Size**
**GSH-PN**	**5.4 ± 1.6 mV**	**5.7 ± 1.4 mV**	**134.1 ± 14.4 nm**
**H_2O_2-PN**	**5.4 ± 1.3 mV**	**11.7 ± 3.4 mV**	**147.1 ± 22.6 nm**
**MAPN**	**5.1 ± 1.8 mV**	**12.3 ± 2.2 mV**	**142.1 ± 16.3 nm**

Table S3. Primers for qRT-PCR quantification of cytosolic mtDNA.

Gene name	Sequence (5′-3′)
D-loop F	CACCAATGCCCCTCTTCTCG
D-loop R	CCTTTCATGCCTTGACGGCT
β-actin F	GGAGATTACTGCCCTGGCTCCTA
β-actin R	GACTCATCGTACTCCTGCTTGCTG
